# NLRP3 Inflammasome‐Mediated Pyroptosis in Osteoporosis: Osteoimmune Mechanisms and Therapeutic Targeting

**DOI:** 10.1111/jcmm.70798

**Published:** 2025-08-25

**Authors:** Jiaxuan Fan, Guokai Du, Te Ba, HuiXin Sun

**Affiliations:** ^1^ Clinical College of Traditional Chinese Medicine Gansu University of Chinese Medicine Lanzhou Gansu P. R. China; ^2^ Sunan Yugur Autonomous County People's Hospital Zhangye Gansu P. R. China; ^3^ Dongfang Hospital Beijing University of Chinese Medicine Beijing P. R. China

**Keywords:** action mechanism, cell pyroptosis, intervention measures, NLRP3 inflammasome, osteoimmunology, osteoporosis

## Abstract

Osteoporosis (OP) is a chronic, age‐related skeletal disorder characterised by progressive bone loss and microstructural deterioration, which increases bone fragility and fracture risk. The NOD‐like receptor family pyrin domain‐containing 3 (NLRP3) inflammasome, a multi‐subunit protein complex involved in bone homeostasis, mediates inflammatory cascades in response to external stimuli and pathological conditions. This process triggers pyroptosis in various bone‐related cells, disrupting bone repair and remodelling. Oestrogen deficiency and aging lead to the overactivation of the NLRP3 inflammasome, stimulate bone immunity, metabolism and other abnormalities and disrupt angiogenesis–osteogenesis coupling. These factors contribute significantly to the pathological progression of OP. However, the precise mechanisms remain poorly understood and are lacking clinical validation. Therefore, this review summarises the mechanisms of NLRP3 inflammasome in response to bone immune signals, external stress and intercellular communication, as well as its role in metabolic regulation, including reprogramming and post‐translational modification, thereby influencing pyroptosis in macrophages, endothelial cells, mesenchymal stem cells and osteoblasts. It explores potential therapeutic strategies that target NLRP3 activation, including exosome (Exos)‐based interventions and traditional Chinese medicine components, which may modulate its differential expression and affect angiogenesis–osteogenesis differentiation. These approaches offer promising avenues for the prevention and treatment of OP.

**Abbreviations**
Akt: protein kinase B
ALP: alkaline phosphatase
AMPK: AMP‐activated protein kinase
Ang‐1: angiopoietin‐1
ASC: apoptosis‐associated speck‐like protein
ATP: adenosine triphosphate
BMMs: bone marrow macrophages
BMSCs: bone marrow mesenchymal stem cells
DDIT3: DNA damage‐inducible transcript 3
ECs: endothelial cells
ER: endoplasmic reticulum
FGF23: fibroblast growth factor 23
GM‐CSF: granulocyte‐macrophage colony‐stimulating factor
GSDMD: Gasdermin D
GSDMs: Gasdermins
HIF‐1α: hypoxia‐inducible factor‐1α
HO‐1: heme oxygenase 1
IFN: interferon
IL: interleukin
LC3 II/I: microtubule‐associated protein 1 light chain 3
LDH: lactate dehydrogenase
LPS: lipopolysaccharide
m^6^A: N6‐Methyladenosine
MDA: malondialdehyde
mtDNA: mitochondrial DNA
mTOR: mechanistic target of rapamycin
MΦs: macrophages
NEK7: NIMA‐related kinase 7
NFATc1: nuclear factor of activated T cells c1
NF‐κB: nuclear factor‐κB
NLRP3: NOD‐like receptor family pyrin domain‐containing 3
Nrf2: Nuclear factor erythroid 2‐related factor 2
OBs: osteoblasts
OCs: osteoclasts
OP: osteoporosis
OPG: osteoprotegerin
OPG: osteoprotegerin
OPG: osteoprotegerin
PD‐L1: programmed death ligand 1
PINK1: PTEN‐induced kinase 1
RANK: receptor activator of nuclear factor‐κ B
RANK: receptor activator of nuclear factor‐κ B ligand
RANKL: receptor activator of nuclear factor‐κ B ligand
ROS: reactive oxygen species
SOD: superoxide dismutase
STAT3: signal transducer and activator of transcription 3
STING: stimulator of interferon genes
TNF‐α: tumour necrosis factor‐α
VDAC: voltage‐dependent anion channels
VEGF: vascular endothelial growth factor

## 1 Introduction

Osteoporosis (OP) is primarily induced by aging, oestrogen deficiency and other pathological factors, leading to increased bone loss and microvascular damage. These changes ultimately disrupt bone homeostasis, increasing bone fragility and fracture risk, with a profound impact on patients' quality of life and physical function. Studies indicate that approximately 200 million people worldwide suffer from OP, affecting one‐third of women and one‐fifth of men over the age of 65, making OP a significant public health burden [1, 2]. The abnormal bone homeostasis induced by the imbalance of bone formation and bone resorption is the root cause of OP [3]; despite advances in understanding, the precise mechanisms underlying OP remain unclear. Recent developments in bone immunology have highlighted the role of NLRP3 inflammasome‐mediated pyroptosis as a key player in bone homeostasis regulation. This process creates a unique immune microenvironment that influences osteoblast function. As a key gene regulating bone metabolism, its polymorphism is closely related to OP genetic susceptibility [4].

Pyroptosis is a form of programmed cell death driven by the Gasdermins (GSDMs) family of proteins. It is characterised by cell swelling, disruption of the plasma membrane and the release of pro‐inflammatory mediators. This process occurs via several mechanisms, including the canonical caspase‐1‐dependent pathway, the caspase‐4/5/11‐mediated atypical pathway and other alternative pathways [5, 6]. Endothelial cells (ECs), bone macrophages (BMMs), bone marrow mesenchymal stem cells (BMSCs) and osteoblasts (OBs) activate the aberrant expression of NLRP3 inflammasome after the initiation of cellular pathogen‐associated molecular patterns (PAMPs) and damage‐associated molecular patterns (DAMPs) upon external stimuli, such as hypoxia and stress, and are involved in the regulation of the pyroptosis signalling pathway, as well as the activation of caspases and GSDMs, promote the release of IL‐1β and IL‐18 and recruit other pro‐inflammatory factors (e.g., leukotrienes, prostaglandins, tumour necrosis factor (TNF‐α), etc.) to generate an inflammatory cascade, which ultimately leads to the focal death of osteoblasts, driving the formation of the inflammatory microenvironment and the aberrant angiogenic–osteogenic coupling [7], which provides new perspectives on the progression of OP pathology; meanwhile, therapies based on the pyroptosis pathway will become a potential choice for the clinical treatment of OP, and cellular pyroptosis mediated by Exos, natural active ingredients, small‐molecule inhibitors and novel materials has shown a unique superiority in the repair of angiogenic–osteogenic coupling in OP. In addition, we innovatively proposed that NLRP3 regulates the ‘nervous system‐vascular‐osteoblast–osteoclast’' axis to participate in OP bone repair. However, the study of the specific mechanism is still preliminary, so in‐depth exploration of the targeting effect of each intervention will help to reveal the underlying molecular mechanism and provide a strategy for regulating bone homeostasis to prevent and treat OP.

## 2 Methodology

This review comprehensively summarises the role of NLRP3 inflammasome‐mediated pyroptosis in osteoporosis and potential therapeutic strategies. The literature search was conducted using electronic databases including PubMed, Web of Science and CNKI, with retrieval terms including ‘NLRP3 inflammasome’, ‘pyroptosis’, ‘OP’, ‘OP‐related complications’, ‘osteolysis’, ‘osteoimmunology’, ‘mechanism’, ‘clinical treatment’ and ‘therapeutic targeting’. Manuscripts with repetitive or incomplete data are excluded. The retrieval time range was from 2020 to the present. Inclusion criteria: (1) original research articles and reviews on the theme of NLRP3 inflammasome, pyroptosis, and OP; (2) molecular mechanisms, signalling pathways or therapeutic interventions related to NLRP3‐mediated pyroptosis in OP.

**FIGURE 1:**
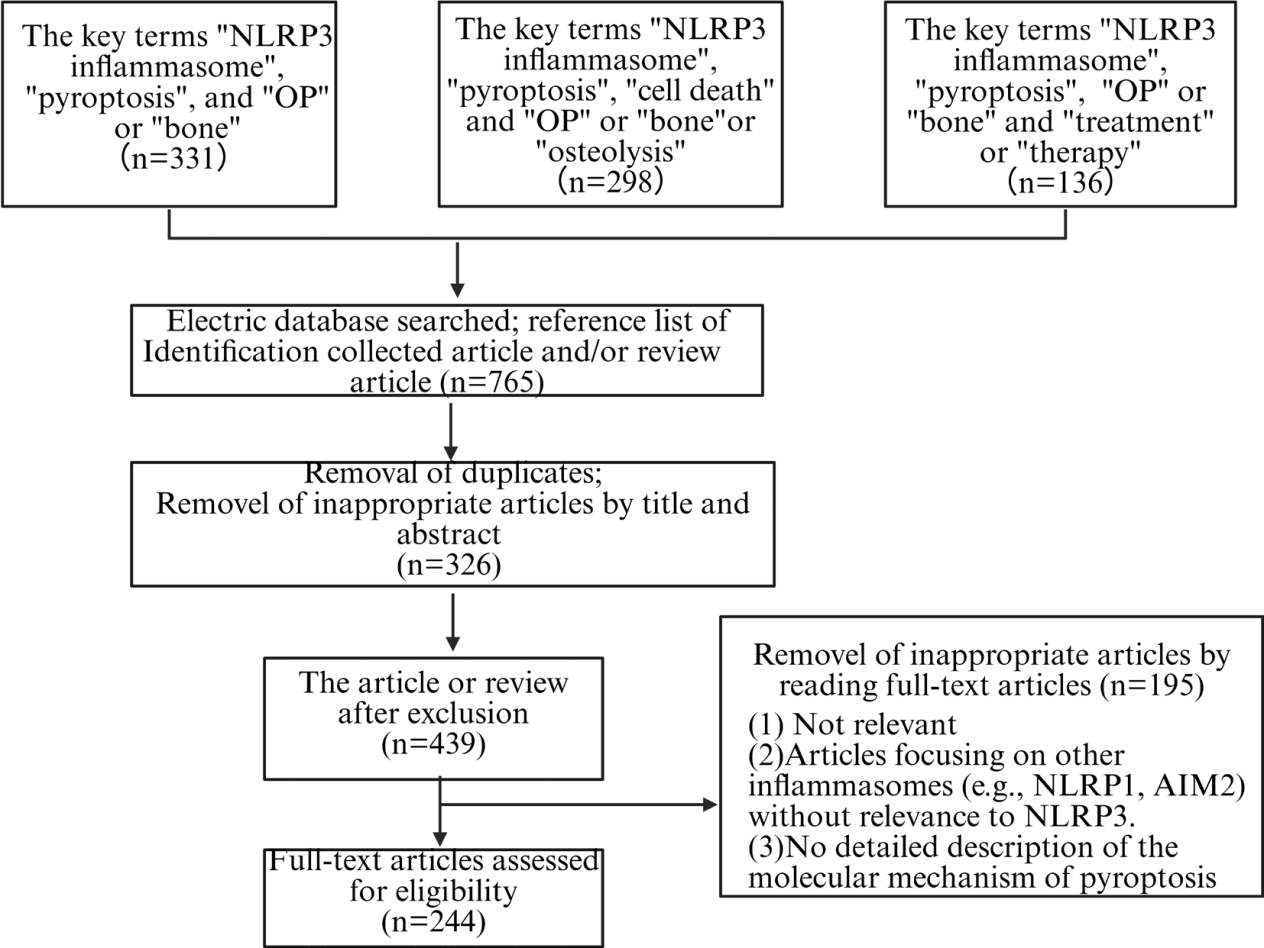
Flowchart summarises the selection process of the research object.

After the initial search, the title and abstract were independently screened by the remaining three authors, and the full text was further evaluated for eligibility by consensus. Finally, this review included 244 relevant articles to support the discussion of mechanisms and treatment strategies (Figure 1).

### 2.1 Targeted Regulation of Pyroptosis by the NLRP3 Inflammasome

NLRP3, the most representative member of the NOD‐like receptor family, consists of a C‐terminal leucine‐rich repeat (LRR) domain, an N‐terminal pyrin domain (PYD) or caspase recruitment domain (CARD) and a central nucleotide‐binding and oligomerisation domain (NACHT) that regulates NLRP3 activity [8]. Activation of NLRP3 recruits downstream apoptosis‐associated speck‐like protein (ASC) and pro‐caspase‐1 to form the NLRP3 inflammasome complex. As a key component of the innate immune system, full expression of the classical NLRP3 inflammasome requires two stages: priming and activation [9]. During the priming phase, PAMPs such as lipopolysaccharide (LPS), viral double‐stranded RNA (dsRNA) and TNF‐α, or DAMPs including reactive oxygen species (ROS), mitochondrial damage and high‐mobility group box 1 (HMGB1), activate toll‐like receptors (TLRs), NOD2 and inflammatory cytokine receptors through ligand–receptor interactions, membrane fusion or endocytosis. This triggers nuclear factor kappa‐B (NF‐κB)‐dependent signalling, leading to the upregulation of precursor components of the NLRP3 inflammasome, including pro‐IL‐1β and pro‐IL‐18 [6]; post‐translational modifications of NLRP3, including phosphorylation and ubiquitination, stabilise it in an autoinhibited conformation. During the activation phase, the NLRP3 inflammasome is triggered by cellular disturbances such as ion flux, reactive oxygen species (ROS) accumulation (particularly mitochondrial ROS (mtROS)), mitochondrial damage, lysosomal disruption and ATP metabolic imbalance, with its unique feature lying in an indirect sensing mechanism – rather than directly binding specific ligands, NLRP3 acts as a broad ‘stress sensor’ by integrating these downstream homeostatic disruptions. This capability enables NLRP3 to unify diverse stimuli (e.g., pathogens, crystalline particles) into a coherent signal reflecting cellular stress and compromised homeostasis [10]. This leads to the activation of caspase‐1, which cleaves pro‐IL‐1β and pro‐IL‐18, while also activating GSDMD to expose its N‐terminal effector domain (GSDMD‐N). Then, it forms pores in the plasma membrane, facilitating the release of IL‐1β and IL‐18 and promoting pyroptosis [11].

**FIGURE 2:**
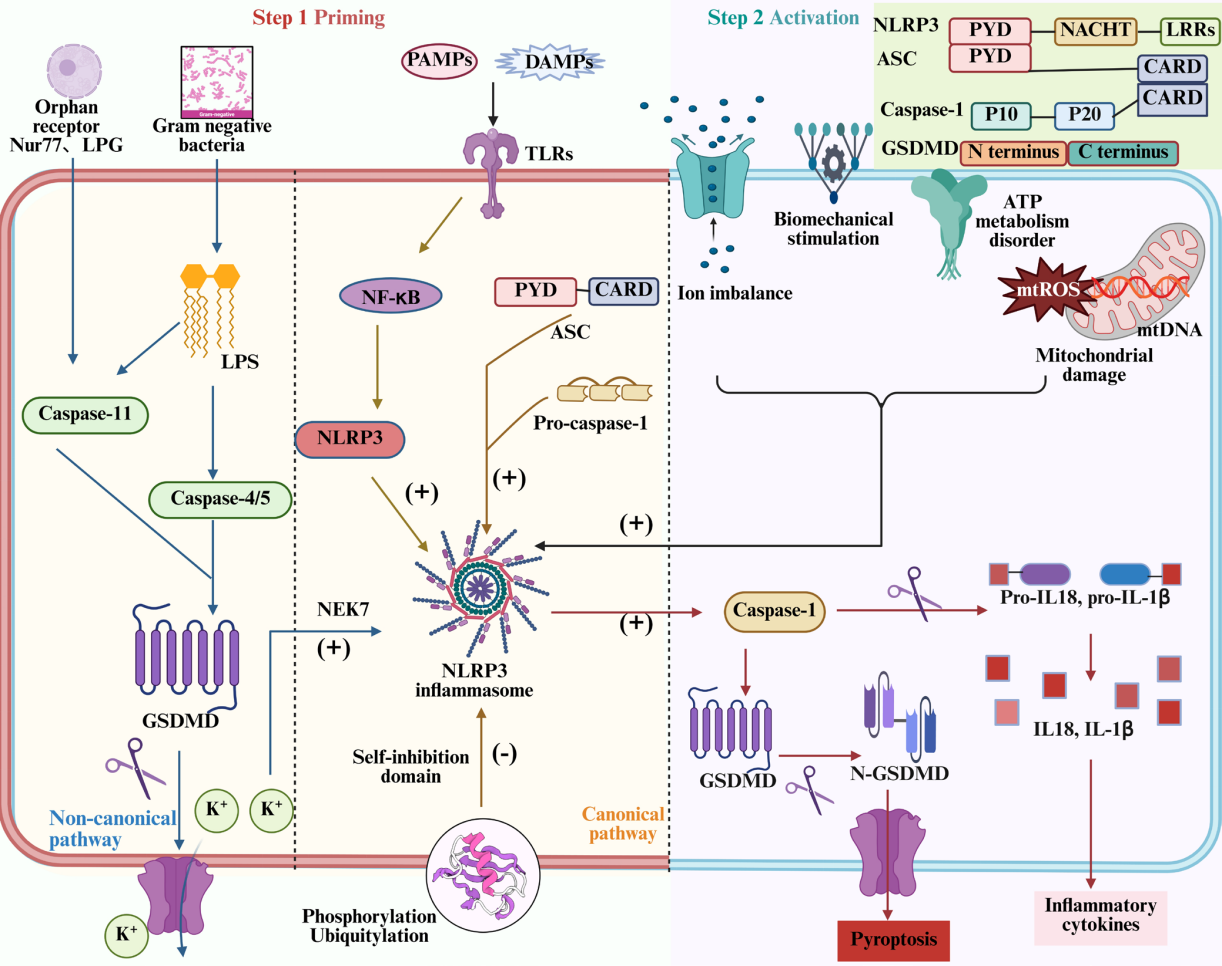
Targeted regulation of pyroptosis by the NLRP3 inflammasome: mechanistic insights. The assembly of the NLRP3 inflammasome is divided into start‐up and activation stages. The first stage contains classical and non‐classical mechanisms involved in pyroptosis. TLRs in the classical pathway sense fine internal and external stimuli, initiate the NF‐κB/NLRP3 cascade reaction to target inflammasome initiation, leading to Caspase‐1 cleavage. In the atypical pathway, LPS activates Caspase‐4/5/11, lyses GSDMD, promotes the production of IL‐1β and IL‐18 and induces NLRP3 inflammasome loading. In the second stage, ion imbalance, mechanical stimulation and abnormal mitochondrial oxidative phosphorylation stimulate Caspase‐1 activation, cleaving pro‐IL‐1β and pro‐IL‐1β into mature forms, promoting downstream inflammatory cascades and pyroptosis.

Human caspase‐4/5 and mouse caspase‐11, key components of the non‐canonical NLRP3 inflammasome‐mediated pyroptosis pathway, directly sense intracellular LPS. Upon activation, they cleave GSDMD, triggering cellular pyroptosis and necrosis, which induces K^+^ efflux. This, in turn, stimulates the expression of NIMA‐related kinase 7 (NEK7), leading to non‐canonical NLRP3 inflammasome activation and promoting the secretion of IL‐1β and IL‐18 [12]. Impaired intestinal barrier upregulates serum LPS levels and induces caspase‐5/11‐GSDMD‐mediated pyroptosis. During Gram‐negative bacterial infection, caspase‐11 responds to LPS transfection, enhances NLRP3 inflammasome activity, interferon (IFN) release and promotes cellular pyroptosis [13]. It suggests that LPS activation of caspase‐4/5/11 cleavage leads to activation of the effector protein GSDMD, which indirectly regulates the expression levels of IL‐1β and IL‐18 and induces non‐classical pyroptosis. This process is crucial for adaptive immunity and cellular stress responses. Meanwhile, orphan receptor Nur77, lipophosphoglycan (LPG) and other factors have been shown to target caspase‐11, activating the non‐canonical NLRP3 inflammasome pathway. However, studies on the specific mechanisms by which these factors contribute to pyroptosis remain limited [14, 15]. Additionally, crosstalk between the classical and non‐canonical NLRP3 inflammasomes significantly enhances the secretion of IL‐1β and IL‐18, enabling cells to better adapt to diverse peripheral environments and external stimuli. This also strengthens the execution of immune responses and the precision of cellular reactions [16, 17]. It is noteworthy that an ‘alternative inflammasome’ pathway in human monocytes, where NLRP3‐ASC‐caspase‐1 signalling occurs without pyroptosome formation or K^+^ efflux dependency, suggests that NLRP3 activation, priming and its downstream effects may be context‐dependent and dissociable events (e.g., the uncoupling of pyroptosis, cell lysis and inflammatory cytokine secretion). Therefore, canonical and non‐canonical NLRP3‐mediated pyroptosis pathways should be further analysed [18]. (Figure 2).

### 2.2 Signalling Pathway Regulates NLRP3 Inflammasome‐Mediated Pyroptosis

#### 2.2.1 NF‐κB/NLRP3 Pathway

NF‐κB, a pleiotropic transcription factor composed of RelA (p65), RelB, p50, p52 and c‐Rel subunits, plays a central role in immune regulation and cell fate. The p50/RelA heterodimer constitutes the canonical NF‐κB complex, tightly regulated by cytoplasmic inhibitors of the IκB family. Upon stimulation via classical (e.g., TLR4, TNF receptor, BCR, TCR) or non‐canonical (e.g., CD40L, BAFF) pathways, IκB undergoes phosphorylation, ubiquitination and subsequent proteasomal degradation, liberating NF‐κB for nuclear translocation and transcriptional activation [19, 20]. A range of upstream signals – including TLRs, mitogen‐activated protein kinase (MAPK) and phosphoinositide 3‐kinase (PI3K)/protein kinase B (Akt) pathways – converge to induce IκB ubiquitination, release the NF‐κB complex and activate the NF‐κB signalling pathway to participate in the pyroptosis cascade.

Aberrant activation of the TLR4/NF‐κB/NLRP3 axis critically contributes to bone degradation and immune dysfunction. Elevated ROS and LPS upregulate TLR4 receptor density and enhance NF‐κBp65 nuclear translocation, amplifying inflammasome signalling. This cascade promotes inflammatory infiltration and pyroptotic cell death, exacerbating tissue injury and skeletal deterioration [21]. Altered phosphorylation dynamics of NF‐κBp65, shifting the p‐NF‐κBp65/NF‐κB p65 ratio, further upregulate NLRP3, IL‐1β and TNF‐α, intensifying pyroptosis and impairing cell migration, thereby sustaining chronic inflammation and tissue dysfunction [22]. Hypoxia‐induced ischemia activates NF‐κB and IκB via TLR4‐dependent mechanisms, driving NLRP3 and IL‐18 expression through multilayered regulation [23]. Conversely, TLR4 demethylation downregulates TLR4, phosphorylated NF‐κBp65 and p‐IκB expression, mitigating NLRP3‐mediated pyroptotic damage [24].

The MAPK pathway – comprising extracellular regulated protein kinases (ERK), p38 and c‐Jun N‐terminal kinase (JNK) – functions via a conserved kinase cascade to activate downstream effectors including NF‐κB, establishing a pivotal pyroptotic signalling hub that coordinates innate and adaptive immunity. Oxidative stress and ischaemia–reperfusion injury induce phosphorylation of p38, ERK and JNK, thereby activating the MAPK/NF‐κB axis, which amplifies NLRP3 inflammasome assembly and pyroptotic cell death, exacerbating inflammatory tissue damage [25]. Elevated glucose levels further potentiate phosphorylation of NF‐κBp65, IκB‐α and ERK1/2, triggering mitochondrial oxidative stress and activating the NLRP3–caspase‐1/3–GSDMD pyroptosis axis in a time‐ and dose‐dependent manner [26]. Additionally, mechanical stress overload converges on the MAPK/NF‐κB/NLRP3 pathway to induce endothelial pyroptosis, linking metabolic and biomechanical insults to inflammatory cell demise [27].

The PI3K/Akt pathway synergises with NF‐κB signalling to amplify immune dysregulation and chronic inflammation. Akt phosphorylates IκB kinase α/β, enhancing NF‐κB activation, while NF‐κB reciprocally upregulates PI3K catalytic subunits, establishing a self‐reinforcing PI3K/Akt/NF‐κB feedback loop that sustains proinflammatory signalling. Elevated ROS, LPS and IL‐1β further potentiate aberrant phosphorylation of NF‐κBp65, PI3K and Akt, collectively intensifying this cascade [28, 29]. Under ischemic, hypoxic and hyperglycaemic conditions, enhanced phosphorylation of these components drives pathological activation of the PI3K/Akt/NF‐κB/NLRP3 axis, triggering the pyroptotic cascade [30, 31].

### 2.3 Piezo1/NLRP3 Pathway

As a mechanosensitive ion channel, Piezo1 transduces mechanical stimuli into electrical signals to regulate cellular functions. It detects aberrant extracellular mechanical forces – including shear and tensile stress – as well as chemical agonists such as Yoda1 and Jedi1/2, disrupting ionic homeostasis and activating the NLRP3/caspase‐1/GSDMD pyroptotic pathway. Concurrently, Piezo1 modulates the Ca^2+^/NF‐κB signalling axis, facilitating NLRP3 inflammasome priming and assembly, thereby driving pyroptosis progression. Elevated shear stress activates Piezo1, triggering Ca^2+^ influx and stimulating the calcium‐activated potassium channel KCNN4 to mediate K^+^ efflux. Additionally, the Piezo1 agonist Yoda1 lowers the activation threshold of the NLRP3 inflammasome, synergistically enhancing NLRP3‐dependent pyroptosis [32]. Overload tensile stress activates Piezo1 signalling, increasing the phosphorylation of NF‐κBp65 and IκB and temporally promoting NLRP3 inflammasome assembly via the Piezo1/Ca^2+^/NF‐κB axis [33]. Ischemia–reperfusion triggers extracellular Ca^2+^ influx mediated by Piezo1 agonists Yoda1 and Jedi1, facilitating Piezo1 sensing of vasodilation‐induced mechanical changes. This cascade elevates p‐NF‐κBp65 and p‐IκBα levels, induces mitochondrial ROS accumulation and oxidative phosphorylation dysfunction, culminating in NLRP3 inflammasome activation [34, 35]. In addition, Piezo1 orchestrates antifungal innate immunity through the Piezo1/Ca^2+^/calmodulin‐dependent kinase/Cyclic adenosine monophosphate response element‐binding protein pathway. This axis induces nuclear transcription factor enhancer binding protein beta (C/EBPβ) expression, upregulates C‐type lectin receptors in innate immune cells, cooperates with NLRP3 inflammasome assembly and amplifies mycelium‐induced pyroptosis cascade damage [36].

### 2.4 JAK/STAT/NLRP3 Pathway

The Janus kinase (JAK)/signal transducer and activator of transcription (STAT) pathway orchestrates inflammatory immune responses, closely linked to pyroptosis effectors including NLRP3, IL‐1β and IL‐18. Under basal conditions, JAK kinases (JAK1, JAK2, JAK3, TYK2) remain inactive until cytokines such as IFNs, TNF and interleukins recruit them to receptor complexes, triggering mutual phosphorylation and activation. Activated JAK phosphorylates STAT proteins (STAT1–6), which dimerise and translocate to the nucleus to regulate target genes, notably NLRP3. Downstream IL‐1β and IL‐18 reinforce this pathway via receptor‐mediated feedback, sustaining pyroptotic signalling and inflammatory amplification. Synergistic stimulation by LPS and IFN‐γ elevates JAK1, p‐STAT1 and p‐STAT3 levels, inducing conformational changes that activate pyroptosis through the JAK/STAT/NLRP3 axis. This interplay fosters a hypoxic microenvironment, enhancing JAK2 and STAT1 phosphorylation, nitric oxide production and NLRP3/caspase‐1/3/8 activation, thereby amplifying pyroptotic inflammation [37, 38]. Similarly, increased phosphorylation of JAK3 and STAT3 upregulates NLRP3 expression and IL‐1β release, while IL‐1β accumulation promotes STAT1 and STAT5 activation, further driving this cascade [39, 40]. Elevated IL‐13 impairs autophagic flux and potentiates JAK1/STAT1/NLRP3 signalling, highlighting the pathway's critical role in pyroptosis regulation during inflammatory pathology [41].

**FIGURE 3:**
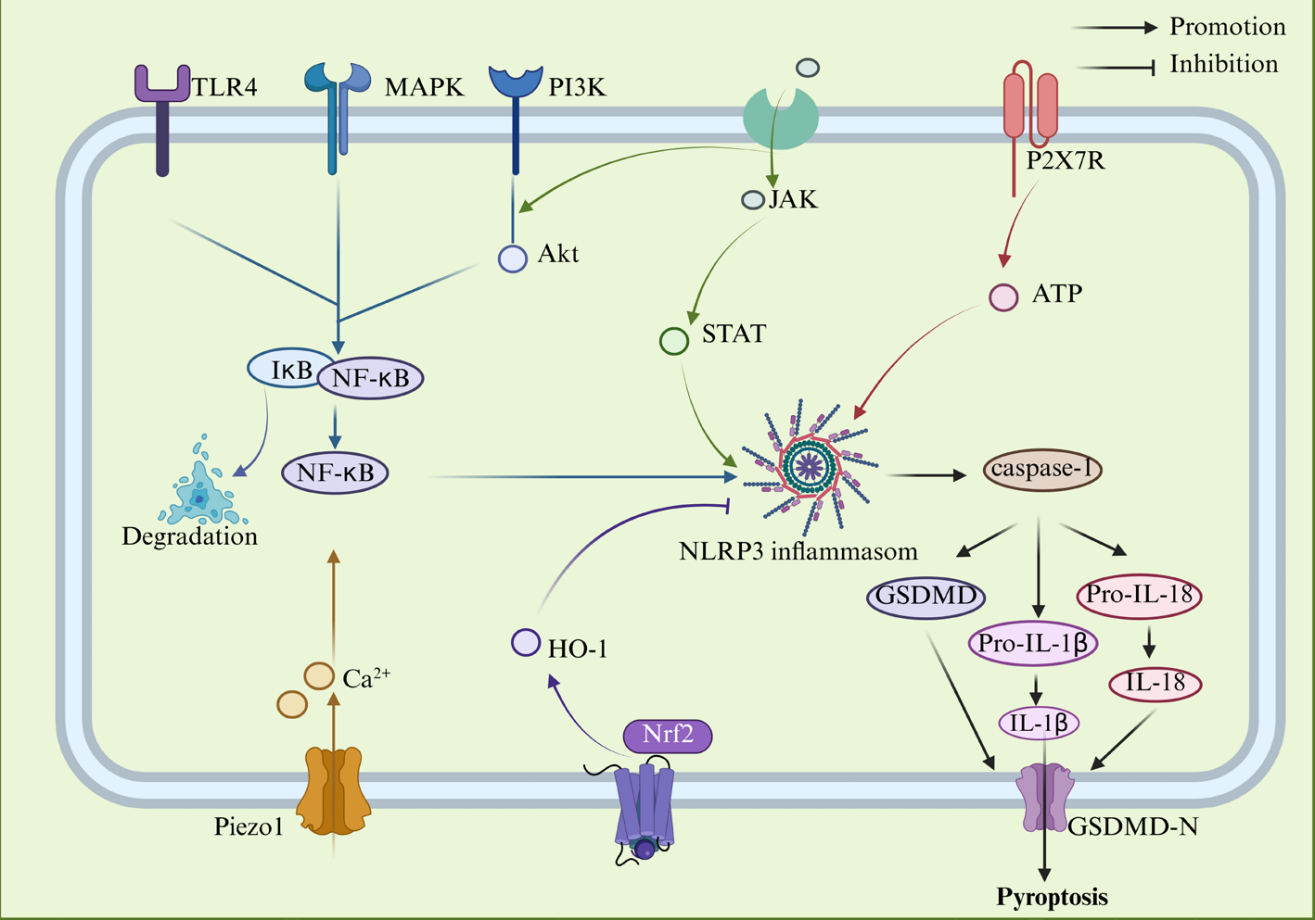
Effect of signalling pathway on NLRP3 inflammasome‐mediated pyroptosis. TLR4, MAPK and PI3K/Akt are common upstream targets of NF‐kB, which promote pyroptosis. Piezo1 affects Ca^2+^ to participate in NF‐kB/NLRP3 signal expression; p2X7R activates ATP to activate NLRP3. JAK positively regulates NLRP3 inflammasome through JAK/STAT and PI3K/Akt pathways. Nrf2/HO‐1 showed an inhibitory effect. To clarify the regulatory effect of different signalling pathways on NLRP3 inflammasome in OP.

In addition, transcription factor‐related factor 2 (Nrf2)/heme oxygenase 1 (HO‐1) is a classic antioxidant and anti‐inflammatory pathway. Upon oxidative stress and endoplasmic reticulum dysfunction, Nrf2 binds antioxidant response elements to induce HO‐1 expression, which negatively regulates NLRP3 inflammasome activation and mitigates pyroptotic damage [42]. Additionally, the Nrf2/HO‐1 axis indirectly suppresses the MAPK/NF‐κB/NLRP3 signalling cascade, reducing the production of pyroptosis mediators [43]. The purinergic receptor P2X7 (P2X7R), an ATP‐gated ion channel, mediates Ca^2+^ and Na^+^ influx alongside K^+^ efflux. Serving as a specific activator of the NLRP3 inflammasome, P2X7R facilitates NLRP3 recruitment, promotes IL‐1β and IL‐18 secretion and accelerates pyroptotic cell death [44] (Figure 3). NLRP3‐mediated pyroptosis is regulated by a complex network of interconnected signalling pathways. Deciphering the precise upstream regulators and downstream effectors of the NLRP3 inflammasome remains imperative to fully understand its modulation and to develop targeted therapeutic interventions.

## 3 Mechanism of NLRP3 Inflammasome Exogenous Regulation‐Mediated Pyroptosis in OP

**FIGURE 4:**
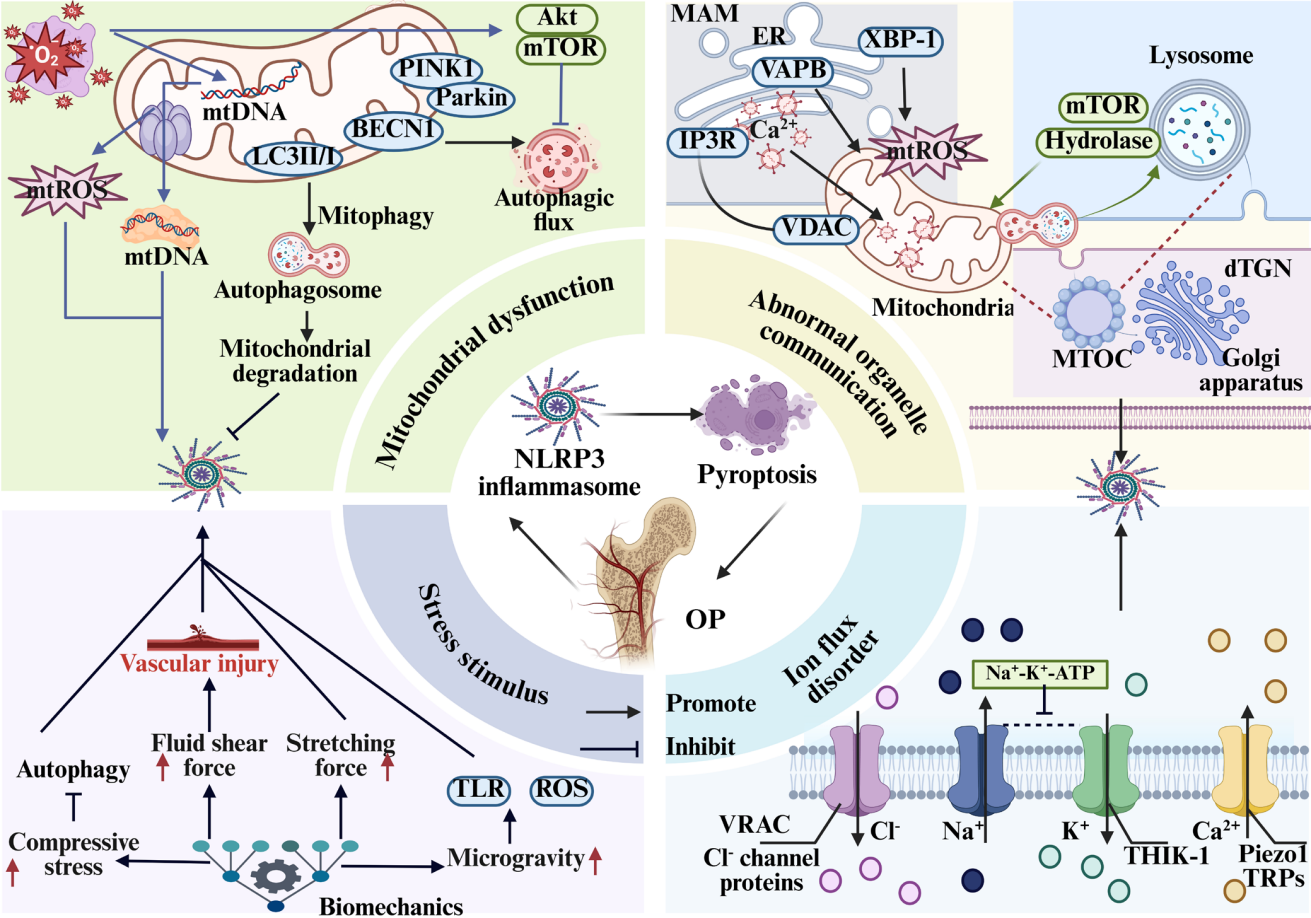
NLRP3 inflammasome‐mediated pyroptosis in osteoporosis: Underlying mechanisms and pathophysiological implications. The activation of NLRP3 inflammasomes, triggered by mitochondrial oxidative stress, metabolic ion dysregulation, abnormal biomechanical cues and disrupted inter‐organelle communication, plays a pivotal role in promoting the pathological progression of OP. Mitochondrial dysfunction – characterised by hypoxia‐induced accumulation of mitochondrial mtROS, cytosolic release of mtDNA and mTOR‐mediated suppression of mitophagy – serves as a key upstream event. Restoration of mitochondrial quality control via upregulation of BECN1, PINK1/Parkin and LC3I/II attenuates this process by repressing NLRP3 activation. Stress stimulus – including prolonged microgravity, excessive compressive or tensile forces and fluid shear stress – potentiate NLRP3 assembly and activation. Abnormal organelle communication – lysosomal hydrolase release and mTOR signalling impair mitochondrial dynamics, while damaged mitochondria initiate autophagosome formation, accelerating lysosomal degradation and NLRP3 activation. ER–mitochondria interactions via VAPB and XBP1 compromise oxidative phosphorylation, while IP3R–VDAC coupling disrupts calcium homeostasis, a potent trigger for inflammasome activation. The Golgi apparatus facilitates spatial coordination of inflammasome assembly through MTOCs and dTGN structures. Ion flux disorder – marked by K^+^ and Cl^−^ efflux, Na^+^ influx and aberrant Ca^2+^ signalling – serves as critical signals for NLRP3 priming and activation.

NLRP3 inflammasome‐mediated cellular pyroptosis is a form of inflammatory programmed cell death driven by caspases. Aberrant activation of this pathway leads to inflammatory bone damage and impaired repair and remodelling, contributing to the development of OP. Additionally, aging and oestrogen deficiency, central to the pathological progression of OP, enhance sensitivity to inflammatory signals in an age‐dependent manner [45]. In the aging‐associated OP mouse model, mutations in the NLRP3 gene trigger aberrant activation of the NLRP3/Caspase‐1/GSDMD axis, leading to skeletal aging and bone loss. In postmenopausal women with OP, inflammatory factors such as NF‐κB and TNF‐α are elevated [46]; silencing of NLRP3 gene expression reduced the levels of pro‐inflammatory factors, including IL‐1β, IL‐6 and TNF‐α and inhibited bone loss. These findings suggest that inflammatory responses mediated by NLRP3 are closely linked to the pathological progression of OP [47]. Meanwhile, mitochondrial dysfunction, bone immunity, mechanical stress stimulation and other effects interact with the NLRP3 inflammasome to respond to the tissue microenvironment to regulate pyroptosis, which is closely related to the pathogenesis of OP (Figure 4).

### 3.1 Mitochondrial Dysfunction

Excessive oxidative stress and inflammation within the bone microenvironment exacerbate cellular dysfunction, mitochondrial DNA (mtDNA) damage and oxidative phosphorylation impairment, resulting in elevated mtROS release that directly and indirectly activates NLRP3‐mediated pyroptosis through feedback regulation. mtROS accumulation induces aberrant opening of the mitochondrial permeability transition pore and voltage‐dependent anion channel (VDAC), facilitating mtDNA accumulation and cytoplasmic escape, activating the IFN gene stimulator pathway, triggering NLRP3 inflammasome‐mediated pyroptosis and inflammatory damage within the bone microenvironment [48, 49]. Simultaneously, mtDNA, as a component of DAMPs, targets the recognition receptor TLR9, activating the NF‐κB pathway and NLRP3 inflammasome, upregulating the levels of IL‐1β, TNF‐α and IL‐6 and triggering pyroptosis [50, 51].

Mitochondrial autophagy synergises with pyroptosis to maintain the bone cell microenvironment, while autophagic dysfunction disrupts this balance, triggering aberrant pyroptosis and contributing to OP‐associated metabolic dysregulation. External stimuli activate the Akt/NF‐κB pathway, promoting mammalian target of rapamycin (mTOR)‐mediated autophagy inhibition [52]. In ischaemia‐, hypoxia‐ and LPS‐induced bone defect models, induction of autophagosome markers Beclin1 (BECN1) restores mitophagy, enhances mitochondrial adaptability and suppresses NF‐κB/NLRP3 signalling. This downregulates caspase‐1, IL‐1β and TNF‐α, mitigating pyroptosis‐driven vascular injury and osteolysis [53]. In OP models induced by high glucose, oestrogen deficiency, or hydrogen peroxide (H_2_O_2_) accumulation, silencing AMPK/mTOR and JAK/STAT3 signalling enhances mitophagy flux, suppresses mtROS release and reduces NLRP3, GSDMD‐N, IL‐18 and IL‐1β expression. This effectively modulates bone immunity, mitigating pyroptosis and preventing bone erosion [54, 55]. Chaperone‐mediated autophagy selectively degrades key proteins, suppressing LPS‐induced NF‐κB activation and NLRP3 inflammasome signalling [4, 16]. Conversely, chronic inflammation impairs mitophagy, disrupts energy metabolism and exacerbates pyroptosis‐driven bone loss. In this inflammatory milieu, Yes‐associated proteins further promote defective mitophagy and NLRP3 activation, amplifying LPS‐induced pyroptosis and cellular senescence, ultimately accelerating osteolysis [40].

### 3.2 Mechanical Stress Stimulation

Excessive or abnormal mechanical stress (microgravity, compressive stress, tensile stress, shear stress, etc.) targets different mechanisms to regulate NLRP3 inflammasome‐mediated pyroptosis and destroy bone metabolism. Research shows that cell–substrate and cell–cell interactions, along with fluid flow stimuli, drive the expression of pyroptosis‐related proteins through mechano‐transduction, enabling cells to sense their microenvironment and mount a controlled response. In contrast, excessive mechanical stress promotes aberrant assembly of the NLRP3 inflammasome, disrupts the adaptation of bone lineage cells to stress and induces an imbalance in bone homeostasis [32]. Long‐term space operations lead to progressive bone loss, the main mechanism of which is the microgravity‐induced decrease in cytoskeletal mechanical properties, stimulation of intracellular ROS production and mitochondrial defects and promotion of NLRP3 inflammasome activation [56]. Simultaneously, microgravity alters the response of bone immune cells, leading to aberrant modifications in the actin cytoskeleton and TLR signalling. This disruption impairs mitochondrial oxidative phosphorylation, upregulates the expression of IL‐1β, IL‐6 and other inflammatory mediators and further exacerbates bone metabolic imbalance through pyroptosis [57]. Skeletal physiology operates within a complex compressive stress environment, where moderate stress inhibits osteoblast pyroptosis under inflammatory conditions, significantly downregulating the levels of NLRP3 and caspases [58]. Conversely, prolonged compressive stress activates the NLRP3/caspase‐1/GSDMD signalling pathway in a time‐dependent manner, resulting in ischemic–hypoxic damage to bone tissue [59]. Excessive compressive force upregulated the mRNA expression levels of NLRP3 and caspase‐1, inhibited autophagy and promoted bone resorption in rat bone tissues. Static compressive stress intervention activated the NLRP3/caspase‐1/IL‐1β axis and induced pro‐inflammatory M1 polarisation, further exacerbating the bone homeostatic imbalance associated with osteoporotic pyroptosis [60]. Stretching forces as a counter effect have also been shown to play an important role in pyroptosis, and early loading of a moderate stretching system effectively downregulated LPS‐induced NF‐κB, NLRP3, TNF‐α and IL‐1β levels and promoted angiogenic–osteogenic coupling [61]. However, prolonged tensile forces disrupt intracellular ion and energy metabolism via the mechanosensitive channel Piezo1, activating NF‐κB signalling and enhancing nuclear translocation. This, in turn, promotes NLRP3/caspase‐1‐mediated pyroptosis and exacerbates bone matrix degradation [33]. Circulatory stretch exacerbates the inflammatory response and ROS accumulation, promotes NLRP3 inflammasome, caspase‐1 expression and induces the generation of pyroptosis, whereas caspase‐1, GSDMD knockdown reverses the injury [62]. Additionally, osteoporotic angiogenesis is modulated by shear stress. Low shear stress suppresses the expression of mechanosensitive microRNAs (miRNAs) in ECs and BMMs, inhibiting the activation of the NLRP3 inflammasome‐dependent pyroptosis pathway [63]; nevertheless, excessive fluid shear stress regulates the Piezo1 channel, stimulating NF‐κB phosphorylation, promoting ASC speck formation and increasing IL‐1β secretion. This triggers cellular pyroptosis, induces abnormal bone immune responses and disrupts vascular maturation and stability [64, 65]. Meanwhile, oscillatory shear force targets and stimulates the expression of multiple mechanosensitive miRNAs, activates NF‐κB and its downstream signalling and promotes pro‐inflammatory ECs factor activation and vascular dysfunction caused by NLRP3 inflammasome [66], suggesting that fluid shear stress regulates cellular focal death involved in OP angiogenic–osteogenic coupling.

### 3.3 Ion Flux Disorder

The transmembrane transport of K^+^, Na^+^, Ca^2+^ and Cl^−^ affects the activation of the NLRP3 inflammasome. K^+^, Cl^−^ efflux, Na^+^ influx overload and Ca^2+^ kinetic abnormalities are key steps in the activation of the NLRP3 inflammasome, leading to ion flux disorder or channel dysfunction, promoting pyroptosis and destroying OP bone homeostasis. K^+^ depletion, a recognised upstream trigger of pyroptosis, leads to mitochondrial depolarisation and excessive assembly of mutated NLRP3 inflammasomes. In contrast, inhibition of K^+^ efflux reduces ROS and mtDNA damage, downregulates NF‐κB and IL‐1β levels and attenuates bone loss induced by pyroptosis [67]. In hypoxic environments, inhibition of the K^+^ channel THIK‐1 reduces NLRP3‐dependent release of IL‐1β and IL‐18, restoring energy metabolism balance. This pathway represents a potential therapeutic target for inflammatory damage [68]. Na^+^ influx, coupled with intracellular K^+^ efflux, regulates NLRP3 activation. Na^+^‐K^+^‐ATPase signalling stabilises the bone's electrophysiological microenvironment, promoting osteocyte proliferation and differentiation [69]. Dysfunction of this mechanism triggers oxidative stress, disrupts energy metabolism and activates the NF‐κB/NLRP3 pathway, thereby facilitating pyroptosis [70]. Deposition of monosodium urate crystals in lysosomes induces intracellular Na^+^ overload, leading to cell swelling and water influx, which reduces intracellular K^+^ levels. This triggers IL‐1β secretion and NLRP3 activation, accelerating inflammatory bone erosion [71]. As a second messenger mediating NLRP3 inflammasome ion mobilisation, aberrant Ca^2+^ dynamics are closely associated with cellular pyroptosis. Activation of Piezo1 ion channels and transient receptor potential (TRP) channels (e.g., TRPV1, TRPM2) promotes Ca^2+^ influx via P2X7R, stimulating NF‐κB/NLRP3 signalling and accelerating pyroptosis‐induced bone erosion [20, 72]. Excessive extracellular Ca^2+^ loading activates calcium‐sensing receptors, promoting the formation of calpain particles and triggering the release of stored Ca^2+^ from the endoplasmic reticulum (ER) and lysosomes. This process induces concentration‐dependent IL‐1β expression, activating the NLRP3 inflammasome and facilitating bone resorption [73]. Mitochondrial Ca^2+^ overload enhances mtROS release and induces mtDNA damage, triggering mitochondrial fission to activate the NLRP3 inflammasome and promote inflammatory bone damage [74]. Additionally, the volume‐regulated anion channel (VRAC) and intracellular Cl^−^ channel proteins influence Cl^−^ efflux, thereby modulating NLRP3 inflammasome activation and contributing to the pathological progression of OP [75].

### 3.4 Abnormal Organelle Communication

Organelles such as mitochondria, endoplasmic reticulum and lysosomes interact physically and through signalling molecules to coordinate the spatiotemporal regulation of the NLRP3 inflammasome, thereby maintaining cellular homeostasis. Recent studies have shown that microtubule organisation centre (MTOC), endoplasmic reticulum membrane (MAM) and ER‐Golgi boundary complex play a key role in their interaction and ensure the core functions of cell energy metabolism, survival and stress environment [76], revealing the key molecules and specific mechanisms of spatiotemporal regulation of OP‐related osteocyte pyroptosis induced by the NLRP3 inflammasome.

#### 3.4.1 Mitochondrial–Lysosomal Interaction

Lysosomes release hydrolases, regulate mTOR signalling and affect mitochondrial dynamics; mitochondrial damage forms autophagosomes and promotes lysosomal degradation. This synergistic effect amplifies the NLRP3 inflammasome‐mediated pyroptosis cascade. Studies have shown that skeletal aging reduces the flux of mitophagy, leading to the accumulation of autolysosomes and undegraded goods and promotes TLR9‐dependent NLRP3 upregulation; at the same time, mtDNA binds and activates TLR9 receptors in lysosomes, induces NF‐κB/NLRP3 expression and aggravates pyroptosis‐induced bone loss [77]. High glucose induces mitochondrial–lysosomal disorders in the skeletal system, promotes the abnormal expression of BECN1, LC3II, BCL2 interacting protein 3 (BNIP3), destroys the ubiquitin‐proteasome system and promotes Sirtuin1(Sirt1)/NF‐κB/NLRP3‐induced musculoskeletal injury [78]. High‐fat diet further increased insulin resistance, promoted mitochondrial‐lysosomal co‐localisation and increased secondary lysosomes and stimulated mtROS‐increased NLRP3 levels [79]. Simultaneously, BMMs exposed to prosthetic wear particles promoted mitochondrial‐lysosomal fusion and mitotic lysosome accumulation and increased BCL2 and BECN1‐mediated NLRP3 inflammasome activation [80]. On the contrary, the lysosomal disruptors Apilimod and Porphyromonas were degraded by Ca^2+^‐dependent lysosomes, resulting in autophagosome‐lysosomal fusion, decreased autophagy flux, induced mtROS and damaged mitochondria accumulation and activated NLRP3 inflammasome [81, 82]. However, inhibiting the lysosomal escape ability can target mitochondria to effectively remove mtROS, revealing the mechanism of mitochondrial‐lysosomal‐mediated NLRP3 inflammasome activation in the pathogenesis of OP [83].

#### 3.4.2 Mitochondria‐Associated Endoplasmic Reticulum Membrane (MAM)

The MAM is involved in regulating autophagy, lipid synthesis and calcium ion transfer, among other processes. It also regulates NLRP3‐mediated pyroptosis. Recently, mitochondrial oxidative stress has caused ER to promote the activation of the NLRP3 inflammasome, resulting in a decrease in bone mass and bone mineral density (BMD) and promoting OP bone loss [84]. Its downstream factor DNA damage‐induced transcription 3 (DDIT3) is repositioned to MAM, which targets Nrf2/NLRP3 signal transduction, upregulates the expression of TNF‐α and IL‐1β and stimulates abnormal bone degradation and remodelling [85]. Ischemia and hypoxia promoted the interaction between the ER transcription protein X‐box binding protein 1 (XBP‐1) and MAM resident protein, promoted caspase‐1‐dependent mitochondrial damage and mtROS release, disrupted ER‐mitochondrial crosstalk and enhanced NLRP3 activation [86]. *Staphylococcus aureus* infection triggers mtROS‐independent mitochondrial damage, which is anchored to MAM by Recombinant Vesicle Associated Membrane Protein Associated Protein B (VAPB), increases the binding between mitochondria, destroys mitochondrial dynamics and promotes NLRP3 inflammasome activation [87]. In addition, the endoplasmic reticulum calcium channel IP3R and mitochondrial VDAC are coupled through chaperone proteins to form calcium transfer microdomains [88]. The continuous inflammatory response leads to MAM remodelling and excessive activation of IP3R in bones, promotes endoplasmic reticulum Ca^2+^ release and mitochondrial Ca^2+^ disorder, stimulates NLRP3 inflammasome assembly [89] and reveals the role of Ca^2+^‐related proteins in OP pyroptosis.

In addition, the Golgi apparatus, as a scaffold for NLRP3 aggregation and assembly activation, interacts with other organelles through the dispersed trans‐Golgi network (dTGN) and MTOCs, and its main role is reflected in the lipid metabolism of NLRP3 in the lower section [76]. Therefore, in the future, we should use organelle‐specific biosensors, super‐resolution microscopy, etc., to reveal the role of organelle communication in regulating NLRP3 in OP pathology. In summary, bone cells sense the surrounding tissue environment and engage in multiple pathways such as oxidative stress, bone immune modulation, autophagy and extracellular stress stimuli to collectively trigger NLRP3‐mediated pyroptosis. Understanding the mechanisms that initiate NLRP3 inflammasome activation is crucial for developing therapeutic strategies aimed at restoring bone homeostasis in OP. However, current research predominantly focuses on cellular and animal models, with large‐scale clinical trials still needed to validate these findings.

## 4 Mechanism of NLRP3 Inflammasome‐Mediated Metabolic Regulation of Pyroptosis in OP

### 4.1 Metabolic Reprogramming

Bone immunometabolism serves as a specific regulatory mechanism for the activation of the NLRP3 inflammasome, with its aberrant metabolic states being closely associated with NLRP3‐mediated pyroptosis [90]. As a sensor of bone homeostasis, NLRP3 inflammasome‐mediated metabolic reprogramming characterises the role of pyroptosis in the progression of OP.

High glycolysis and low oxidative phosphorylation promote the activation of the NLRP3 inflammasome. Glycolysis converts glucose into pyruvate and ATP, providing energy for bone repair and remodelling. Meanwhile, the activation of glycolysis and the cytoplasmic accumulation of its metabolic products, such as glucose‐6‐phosphate and lactate, accelerate the assembly of the NLRP3 inflammasome and promote pyroptosis. Studies have shown that in hypoxia‐induced inflammatory injury models of BMSCs, activation of the PI3K/AKT/mTOR signalling pathway enhances cellular glycolysis, activates the NLRP3 inflammasome and promotes an inflammatory cascade [91]. Lysine acetyltransferase 2A can inhibit the activity of Nrf2 and the expression of downstream antioxidant factors, thereby promoting glycolytic reprogramming and activating the NLRP3 inflammasome to cause inflammatory bone resorption [92]. In human BMMs treated with triclosan, mitochondrial respiration is reduced, leading to a shift from oxidative phosphorylation to glycolysis. This metabolic reprogramming activates the NLRP3 inflammasome and triggers the pyroptosis cascade [93]. Glucose transporter 1 (Glut1), the primary transporter regulating glucose uptake, promotes glucose uptake and glycolysis in an inflammatory microenvironment, rapidly upregulating NLRP3 expression [94]. Conversely, knocking down Glut1 levels reverses NLRP3 inflammasome assembly and glycolytic activation, promoting oxidative phosphorylation and osteogenic differentiation [95]. Overexpression of pyruvate dehydrogenase kinase impairs mitochondrial autophagy flux, increases mtROS levels and promotes reprogramming of the NLRP3 inflammasome [96]. In contrast, inhibition of the mitochondrial pyruvate carrier suppresses glycolysis and mitochondrial lactate production, thereby inhibiting NLRP3 inflammasome‐induced inflammatory bone loss [97]. This study elucidates the involvement of glycolysis in the initiation of NLRP3 inflammasome‐mediated pyroptosis and its disruption of bone homeostasis in OP.

TCA cycle, such as itaconate, succinate and fumarate, has been shown to indirectly participate in NLRP3 expression. They promote energy metabolism and angiogenesis–osteogenesis coupling and regulate pyroptosis‐induced OP bone resorption. Studies have shown that itaconate modifies cysteine residue C548 on NLRP3, disrupting the interaction between NLRP3 and its target NIMA‐related kinase 7. This modification inhibits the expression of NF‐κB/NLRP3/caspase‐1 and suppresses pyroptosis [98]. Concurrently, itaconate effectively inhibits NLRP3 inflammasome‐dependent IL‐1β expression, thereby ameliorating extracellular matrix degradation and inflammatory bone loss [99]. Conversely, activation of VRAC promotes the efflux of itaconate and the accumulation of mtROS, thereby activating the NLRP3 inflammasome and accelerating swelling and pyroptosis in BMSCs [100]. Additionally, mitochondrial Ca^2+^ signalling regulates mitochondrial metabolism by targeting key enzymes of the TCA cycle. Its unidirectional transport can respond to LPS and TLR signalling, promoting pyroptosis in BMMs induced by NLRP3, IL‐1β and IL‐6. Fumarate can block NLRP3 assembly, oligomerisation and pyroptosis [101]. Its analogue, fumarate ester, inhibits NF‐κB/NLRP3 activation in human BMMs, thereby suppressing inflammatory responses and mtDNA damage. Conversely, succinate and its oxidation effect can promote IL‐1β expression [102]. Succinate accumulation establishes a localised hypoxic microenvironment that activates the NLRP3 inflammasome, enhancing the production of IL‐1β, IL‐6 and TNF‐α. As a TCA cycle intermediate, succinate also facilitates IL‐1β maturation and NLRP3 inflammasome activation in rat bone marrow‐derived BMMs, promoting pyroptosis and inflammatory bone erosion. These findings implicate succinate as a key metabolic driver of osteoporotic pathogenesis [90, 103].

Lipid metabolism characterises the selective assembly process of the NLRP3 inflammasome. Saturated fatty acids (FAs) activate the NLRP3 inflammasome, whereas polyunsaturated FAs tend to inactivate it. Excessive lipid intake increases circulating FA levels, leading to metabolic disorders, ageing and obesity‐associated OP. Studies have shown that diets rich in saturated FAs lead to mitochondrial membrane potential disruption and accumulation of mtROS and lactate dehydrogenase (LDH). These changes promote the activation of the Sirt1/NLRP3 signalling pathway, which in turn enhances GSDMD‐mediated pyroptosis in ECs in a dose‐dependent manner [104]. This process can disrupt the coupling between angiogenesis and osteogenesis. Concurrently, in a murine model of post‐traumatic bone resorption with obesity, a diet rich in saturated FAs promotes the expression of TLR4/NF‐κB and NLRP3/caspase‐1/GSDMD signalling pathways [22]. In contrast, a diet rich in n‐3 polyunsaturated FAs inhibits these pathways and pyroptosis‐induced bone loss [105]. Additionally, short‐chain fatty acids can inhibit the NLRP3/caspase‐1/GSDMD axis, thereby downregulating the levels of IL‐1β, IL‐18 and ASC and ameliorating pyroptosis. FA synthesis and oxidation have been shown to stimulate NLRP3 expression. A high‐fat diet upregulates carnitine palmitoyltransferase 1A, a key enzyme in FA oxidation induced by palmitate, thereby enhancing NLRP3/caspase‐1/GSDMD expression and accelerating pyroptosis and atrophy in skeletal muscle [106]. However, the underlying mechanisms still need to be further explored.

**FIGURE 5:**
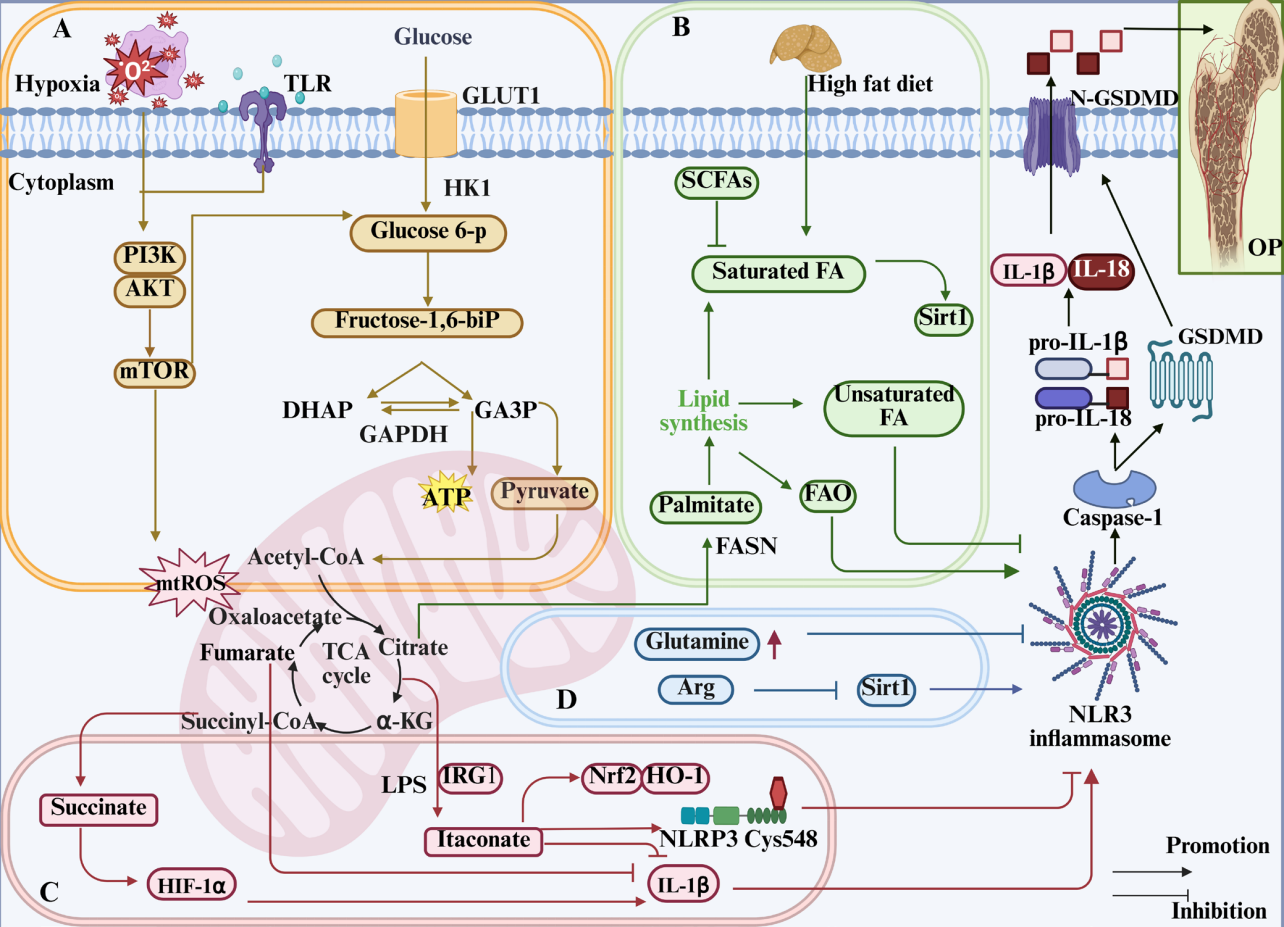
Role of NLRP3 metabolic reprogramming‐mediated pyroptosis in OP. Glycolysis, TCA cycle, lipid and amino acid metabolism are involved in the reprogramming of NLRP3 metabolism, regulating the assembly of inflammasomes and targeting the role of pyroptosis in OP. (A) Glycolysis process. The uptake and decomposition of glucose is the basis of glycolysis. Extracellular glucose is transported into the cytoplasm through Glut1 and chemically reacts to decompose to form pyruvate, which then diffuses into mitochondria and participates in the TCA cycle. At the same time, hypoxia stimulation and TLRs expression trigger mTOR and hexokinase 1 (HK1)‐dependent glycolysis and promote NLRP3 inflammasome activation. (B) Lipid metabolism process. Citric acid in mitochondria accumulates and transports to the cytoplasm, activating fatty acid synthase (FASN) as a key signalling molecule, promoting the synthesis of fatty acids and then participates in the regulation of NLRP3 inflammasome. Saturated FA and fatty acid oxidation (FAO) activate NLRP3 inflammasome, while unsaturated FA inhibits its assembly and activation. (C) TCA cycle process. Succinate accumulates in mitochondria, stabilises HIF‐1α expression and releases IL‐1β to promote NLRP3 activation. Fumarate showed an inhibitory effect. Itaconation is synthesised by the decarboxylation of cis‐aconitic acid by the immune response gene 1 (IRG1), which promotes Nrf2/HO‐1 expression by alkylation of NLRP3 cysteine residues to inhibit NLRP3 transcription. (D) Amino acid metabolic process.

Recently, the metabolism of amino acids such as arginine and glutamine has provided new insights into the activation of the NLRP3 inflammasome. High glutamine metabolism specifically inhibits NLRP3 inflammasome assembly and also promotes the TCA cycle to enhance effector functions [107]. In contrast, glutamine deprivation downregulates endogenous itaconate levels, accelerates pyroptosis and promotes the maturation of IL‐1β and IL‐18. Arginine (Arg), an essential amino acid, has been shown to inhibit Sirt1/NLRP3 signalling stimulated by LPS/ATP, thereby improving pyroptosis in ECs and promoting bone vascular maturation in OP [108]. This finding reveals that amino acid metabolism can modulate the NLRP3 inflammasome to improve bone formation in OP (Figure 5).

### 4.2 NLRP3 Post‐Translational Modification

Post‐translational modifications (PTMs) such as methylation, phosphorylation, ubiquitination and acetylation are distinct from metabolic reprogramming, chemically modify NLRP3 and influence its interactions with adaptor proteins ASC and pro‐Caspase‐1 [76]. By enhancing its intracellular stability and precise expression, these modifications offer new insights for translating findings from animal models into clinical prevention and treatment of OP.

NLRP3 is ubiquitinated in its resting state and deubiquitinated upon activation; ubiquitin E3 ligases and deubiquitinating enzymes can be targeted for the treatment of OP. Studies have shown that the E3 ubiquitin ligase TRIM31 targets K48‐linked polyubiquitination of NLRP3, promoting its ubiquitination and degradation. This process improves endothelial dysfunction and bone vascular repair [109]. In a mouse model of bone defect, inhibiting the colocalisation of ubiquitin carboxyl‐terminal hydrolase 5 with NLRP3 maintains the stability of the ubiquitin–proteasome system in BMSCs, accelerating NLRP3 ubiquitination to promote osteogenic differentiation [110]. In hypoxic conditions, the deubiquitinating enzyme ubiquitin‐specific peptidase (USP)14 targets and binds to the leucine‐rich repeat (LRR) domain of NLRP3, inhibiting NLRP3 ubiquitination levels. This action accelerates pyroptosis in BMSCs and inflammatory bone resorption [111]. In a high‐glucose environment, overexpression of USP30 promotes NLRP3 deubiquitination, activates the NLRP3/caspase‐1 p20 signalling pathway and upregulates the levels of IL‐1β and IL‐18. These findings suggest that it may induce OP associated with diabetes [112]. Additionally, in a murine model of arthritis, USP16 promotes mitochondrial fission in a dynamin‐related protein 1 (Drp1)‐dependent manner, accelerating NLRP3 ubiquitination and stabilisation, thereby inducing bone resorption [113].

Palmitoylation modification localises NLRP3 to the trans‐Golgi network or the microtubule‐organising centre, thereby promoting the pyroptosis cascade. TLRs promote the palmitoylation of specific cysteine residues on NLRP3, translocating it to dispersed trans‐Golgi network vesicles and accelerating its activation and assembly. Palmitate competitively binds to these sites, thereby inhibiting inflammatory bone resorption [114]. Zinc‐finger DHHC18 catalyses NLRP3 palmitoylation, recruiting it to the microtubule‐organising centre and enhancing the positive feedback of membrane trafficking on inflammasome assembly, thereby accelerating pyroptosis‐mediated damage in OP [115]. Additionally, NLRP3 methylation is carried out by methyltransferases, including protein lysine methyltransferases and protein arginine methyltransferases, which transfer a methyl group from S‐adenosylmethionine to specific residues on NLRP3. This process promotes NLRP3 methylation and assembly, thereby accelerating glucocorticoid‐induced bone damage in OP [116]. In BMMs, inflammasome degradation is regulated by phosphorylation of the LRR domain of NLRP3, which is dephosphorylated upon activation [117]. Elucidating the bidirectional regulatory mechanisms of PTMs on NLRP3 inflammasome assembly and activity allows cells to more precisely control their metabolic states, offering new perspectives for precision treatment of OP.

A recent study demonstrated that NLRP3 palmitoylation and hexokinase 2 act synergistically to enhance mitochondrial metabolic reprogramming, thereby promoting inflammasome activation and pyroptosis [118]. This finding reveals that PTMs and metabolic reprogramming interact through the activities of metabolic enzymes, substrates and cofactors to regulate cellular metabolism. This fine‐tuning of metabolic states enables cells to adapt to various physiological and pathological environments. However, such research is rarely seen in the context of OP.

## 5 Role of NLRP3 Inflammasome‐Mediated Pyroptosis in OP

OP arises from dysfunctional coordination among skeletal cell types. BMSCs, ECs and OBs collaborate to establish bone vasculature and refine the osseous microstructure, while OCs derived from haematopoietic lineages mediate resorption of aged or damaged bone matrix. Excessive pyroptosis impairs angiogenesis–osteogenesis coupling in ECs and OBs, while augmenting OCs‐driven bone resorption, thereby disrupting skeletal homeostasis. Concurrently, osteocytes fail to transduce mechanical cues and maintain ionic balance, and aberrant osteoimmunological signalling exacerbates bone mass loss. Crucially, cell‐type‐specific functions, phenotypes, pyroptotic pathway activation states and sensitivity thresholds engender distinct mechanistic roles for pyroptosis across OP pathogenesis.

### 5.1 NLRP3 Inflammasome Involvement in Macrophage Pyroptosis

Macrophages(MΦs), as pivotal immune cells residing in the skeletal and vascular microenvironment, exhibit dual roles in both local tissue homeostasis and innate immune regulation. They are broadly classified into classically activated pro‐inflammatory M1 macrophages and alternatively activated anti‐inflammatory M2 macrophages. M1 polarisation is induced by LPS, interleukins (IL‐1, IL‐2, IL‐6, IL‐8, etc.), IFN‐γ and granulocyte‐macrophage colony‐stimulating factor (GM‐CSF), leading to the secretion of pro‐inflammatory mediators such as TNF‐α, IL‐12, NO and ROS, thereby exacerbating chronic inflammatory responses. In contrast, M2, as a selectively activated cell, is activated by IL‐4, IL‐10, TGF‐β and macrophage colony‐stimulating factor (M‐CSF) in the late stage of inflammation, and paracrine pro‐angiogenic factors (vascular endothelial growth factor (VEGF), etc.) improve angiogenesis–osteogenesis coupling. The dynamic balance of M1/M2 polarisation, along with the regulation of NLRP3 inflammasome assembly, is critical for maintaining bone immune homeostasis and presents a promising therapeutic target for OP. Additionally, MΦ‐derived immunomodulatory factors can drive their differentiation into osteoclasts (OCs), further influencing bone remodelling and OP pathophysiology [54, 65].

Emerging evidence from bisphosphonate‐induced osteonecrosis models suggests that P2X7R/ROS/NLRP3 signalling facilitates M1 macrophage polarisation, exacerbating the inflammatory microenvironment. On the contrary, upregulation of IL‐37 promotes M1 pyroptosis, thereby mitigating inflammatory bone damage and potentially offering a novel therapeutic strategy for osteonecrosis‐related bone loss [119]. In LPS‐induced osteolysis models, activation of the NF‐κB/NLRP3 signalling pathway upregulates inducible nitric oxide synthase (iNOS) and IL‐1β while suppressing Arg1 expression. This shift drives pyroptosis of CD206^+^ M2 macrophages, disrupting the pro‐regenerative immune balance and exacerbating OP bone defects [120]; conversely, fused extracellular vesicles derived from M2 macrophages and BMSCs attenuate ROS accumulation, reduce the IL‐1β/IL‐18 ratio and inhibit caspase‐1‐dependent M2 pyroptosis, thereby enhancing trabecular bone volume and density [121]. Under hypoxic conditions, MΦ glycolytic reprogramming enhances ATP release, activating the AMPK/NF‐κB signalling axis and driving the expression of IFN‐γ, GSDMD and IL‐1β. This cascade exacerbates M2 pyroptosis [122]. Methyltransferase 3 (METTL3), as an upstream regulator of NLRP3, facilitates its post‐translational modification by targeting N6‐methyladenosine (m^6^A) under hyperglycaemic conditions. This modification enhances NLRP3 mRNA stability, leading to the upregulation of caspase‐1, GSDMD and IL‐1β, thereby promoting M2 pyroptosis and impairing osteogenic differentiation [123]. Short‐chain fatty acids, metabolites of the gut microbiota, reprogram NLRP3 through lipid metabolism, oligomerise ASC spots, inhibit M2 pyroptosis and OCs formation and reduce wear particle‐induced osteolysis [124]. Furthermore, joint replacement following OP fractures increases prosthesis‐derived wear particles, disrupting mitochondrial autophagy in MΦ. This process triggers mtROS‐induced M2 pyroptosis, accelerates periprosthetic M1 infiltration and OCs differentiation and ultimately exacerbates bone resorption and prosthetic loosening [125]. In BMMs derived from the tibia and femur of mice, overexpression of LDH enhanced ASC oligomerisation and induced the expression of caspase‐1 and IL‐1β in response to ATP, monosodium urate. Subsequently, it triggered pyroptosis, thereby promoting osteolysis in OP [126]. Defects in volume‐activated Cl^−^ channels facilitate the persistent activation of NLRP3/caspase‐1 signalling in response to LPS, leading to GSDMD cleavage and its interaction with inositol phosphate in the plasma membrane, resulting in BMMs' oedema and cracking [127]. Furthermore, mechanical stimuli such as compression and fluid shear stress activate NLRP3 inflammasome assembly, thereby accelerating BMMs' pyroptosis and driving bone loss in OP [59, 128].

### 5.2 NLRP3 Inflammasome Involvement in Endothelial Cells Pyroptosis

ECs, characterised by high expression of platelet‐endothelial cell adhesion molecule (CD31) and endothelial membrane protein (EMCN), serve as ideal progenitor cells for repairing H‐type vessels and maintaining bone homeostasis in OP. They respond to signalling pathways, including hypoxia‐inducible factor‐1α (HIF‐1α)/VEGF, vascular cell adhesion molecule 1 (VCAM1) and bFGF, to form a complete vascular network, providing structural support and stable pathways for bone cells. As upstream regulators of bone formation, ECs secrete osteogenic factors such as bone morphogenetic proteins (BMPs), osteoprotegerin (OPG) and alkaline phosphatase (ALP), which recruit osteoprogenitor cells to damaged sites. They also supply essential nutrients, oxygen and deliver multipotent cells and minerals, thereby promoting the coupling of angiogenesis–osteogenesis [129]. However, aging, oestrogen deficiency, glucose metabolism disorders and prolonged hormone use contribute to the overexpression of NLRP3 protein, thereby impairing H‐type vascular repair and disrupting bone remodelling in OP [4].

In a mouse model of inflammatory bone defects induced by LPS and ATP, immunofluorescence co‐localisation revealed a significant downregulation of CD31, VEGFA and angiopoietin‐1 (Ang1), key regulators of angiogenesis; the elevated expression of GSDMD‐N and IL‐1β promoted ECs cleavage and pyroptosis [130]. Simultaneously, LPS combined with methylprednisolone induced fat accumulation around the tibia in mice, upregulated NLRP3 and IL‐6 protein levels and suppressed VEGF and VCAM1 expression. This cascade promoted endothelial cell pyroptosis and H‐type vascular injury, leading to impaired bone mineralisation and reduced trabecular density, thereby exacerbating bone fragility [131]. In the oxidative damage model of ECs induced by H_2_O_2_, the enrichment of LDH and mtROS reduces the activity of Nrf2, HO‐1 and superoxide dismutase (SOD), increases the ratio of LC3II/I specifically bound to the autophagosome membrane, stimulates the occurrence of abnormal mitophagy, upregulates the secretion levels of NLRP3 and IL‐1β and promotes the senescence and pyroptosis of ECs [132]. Meanwhile, the ischemic microenvironment suppresses the expression of transcription factor EB, modulates the AMPK/mTOR signalling pathway to reduce autophagic flux and facilitates the accumulation of ROS. This cascade leads to the upregulation of NLRP3 and IL‐1β expression in ECs, exacerbating ECs' pyroptosis following ischemia–reperfusion injury [133]. Overexpression of programmed death ligand 1 (PD‐L1) in ECs disrupts mitochondrial oxidative phosphorylation, triggering the activation of the mtROS/NLRP3/caspase‐1 signalling cascade. This pathway inhibits ECs' proliferation and homing, ultimately impairing angiogenesis–osteogenesis coupling in OP [134]. Simultaneously, FGF23 suppresses the activation of the HIF‐1α/VEGF axis under hypoxic conditions, leading to increased expression of pro‐inflammatory cytokines IL‐1β, IL‐6 and TNF‐α, while downregulating VEGF, CD31 and ALP protein levels. This cascade promotes the assembly of the NLRP3 inflammasome, disrupting H‐type vascularisation and osteogenic differentiation, ultimately impairing bone homeostasis [135]. METTL3 catalyses m^6^A modification to enhance NLRP3 expression, promote ROS accumulation, elevate IL‐1β and IL‐18 levels and exacerbate LPS/ATP‐induced pyroptosis in ECs. Conversely, ECs‐derived Exos enriched with nuclear‐enriched abundant transcript 1 mitigate this injury by suppressing the NF‐κB/DEAD‐box helicase 3 X‐linked (DDX3X)/NLRP3 signalling axis, upregulating CD31, VEGF, ALP and BMP expression and thereby promoting angiogenesis and osteogenic differentiation [136, 137], highlighting that various target proteins mediated ECs pyroptosis can be involved in the pathological process of OP. Moreover, prolonged microgravity induces endoplasmic reticulum stress in ECs, facilitating Ca^2+^ transfer to mitochondria, triggering mitochondrial fission, mtROS accumulation and PTEN‐Induced Kinase 1( PINK1)/Parkin‐dependent mitophagy. This cascade activates the iNOS/NO/NF‐κB/NLRP3 inflammasome pathway, suppresses VEGFA and VCAM1 expression and results in increased ECs' pyroptosis [19, 138].

### 5.3 NLRP3 Inflammasome Involvement in Bone Marrow Mesenchymal Stem Cells Pyroptosis

The proliferation and differentiation of BMSCs play a pivotal role in the pathogenesis of OP. External oxidative stress and inflammation abnormally activate the NLRP3 inflammasome, disrupting the balance between osteogenic and adipogenic differentiation in BMSCs, thereby accelerating bone loss. Studies in an ovariectomy (OVX)‐induced OP mouse model have shown that NLRP3, caspase‐1, IL‐1β and IL‐18 expression is upregulated, while the expression of Runt‐related transcription factor 2 (Runx2), Osterix and osteopontin (OPN) is suppressed. This inverse relationship between the impaired osteogenic potential of BMSCs and bone loss highlights a key mechanism underlying OP progression [139]. Conversely, cylindromatosis stabilises Runx2, OPN and osteocalcin (OCN) levels in BMSCs via the deubiquitination pathway, inactivates the NLRP3 inflammasome and mitigates OVX‐induced bone loss and structural deterioration [140]. In a cigarette smoke‐induced OP mouse model, compressive stress significantly reduces the femur's maximum bending load. Meanwhile, NLRP3, GSDMD and IL‐1β expression in BMSCs increases in a concentration‐dependent manner, while Runx2, OPN and type I collagen α1 (Col1a1) levels are downregulated. This dysregulation induces BMSCs' pyroptosis and disrupts bone immune homeostasis. Notably, the pyroptosis inhibitor MCC950 effectively reverses these pathological changes [141]. Excessive compressive stress enhances TNF‐α and IL‐6 secretion in bone tissue, activating NF‐κB/NLRP3 signalling in BMSCs. This cascade induces mTOR‐mediated mitophagy and mtROS release, ultimately impairing the osteogenic differentiation of BMSCs [142]. A vertical mechanical load of 20 N activates the Piezo1–Ca^2+^ ion channel. Transcriptomic analysis indicates that this activation promotes the expression of the PI3K/AKT signalling pathway, upregulates the levels of NLRP3, IL‐6, IL‐1β and TNF‐α and enhances pyroptosis and abnormal osteogenesis in BMSCs. Additionally, various metabolic products of BMSCs are involved in regulating pyroptosis [143]. Staphylococcal protein A induces inflammatory femoral defects and bone loss by activating the MAPK/NLRP3 signalling axis, promoting BMSCs' pyroptosis and inhibiting osteogenic differentiation [144]. As an NLRP3 inflammasome agonist, advanced glycation end products enhance NLRP3 and caspase‐1 secretion within the inflammatory microenvironment, drive adipogenic differentiation of BMSCs and contribute to ageing‐related OP [145]. Furthermore, BMSCs' pyroptosis plays a crucial role in osteoporotic fracture repair. In a femoral condyle defect model, prolonged implantation of allograft bone scaffolds induces overexpression of nerve growth factor and the long non‐coding RNA interferon response negative regulator, activating NF‐κB/NLRP3 signalling. This upregulates GSDMD and IL‐1β mRNA levels while suppressing OCN and BMP gene expression, ultimately accelerating BMSCs membrane rupture and pyroptosis [146, 147]. A high‐fat diet induces the formation of neutrophil extracellular traps and abnormal lipid metabolism of the NLRP3 inflammasome, suppressing the expression of ALP, OCN, Runx2, VEGF, CD31 and others. This action inhibits angiogenesis–osteogenesis coupling and delays fracture healing in OP [148].

### 5.4 NLRP3 Inflammasome Involvement in Osteoblasts Pyroptosis

NLRP3 inflammasome‐mediated signalling pathways inhibit OBs‐driven bone formation under various bone turnover conditions, including oestrogen deficiency and hyperglycemia. Notably, NLRP3 knockout alleviates bone loss in high bone turnover models, highlighting its potential as a therapeutic target for OP. In an LPS/ATP‐induced inflammatory osteolysis model, activation of the NLRP3/caspase‐1/GSDMD signalling pathway led to a time/concentration‐dependent increase in LDH and ROS release, along with elevated secretion of IL‐6, TNF‐α and IL‐1β. Concurrently, the expression of Runx2, ALP and Col1a1 was suppressed, exacerbating oxidative stress/inflammation‐induced pyroptosis of OBs [149]. Notably, MCC950 effectively reversed these effects, promoting OBs' proliferation and differentiation [150]. Sodium butyrate accelerates progressive bone loss in mice by activating the caspase‐3/GSDME signalling pathway, promoting LDH release and enhancing NLRP3 inflammasome assembly. This disrupts the OPG/Receptor Activator of Nuclear Factor‐κ B Ligand (RANKL) axis, leading to OBs dysfunction and impaired bone homeostasis [151]. Excessive TNF‐α exposure activates the NLRP3/caspase‐1/3 axis, promoting GSDMD/GSDME‐mediated plasma membrane cleavage and upregulating IL‐1β and IL‐18 expression. Concurrently, it downregulates OPN and OCN levels, exacerbating OBs' pyroptosis and inflammatory bone damage [152]. Oestrogen deficiency compromises the intestinal barrier and exacerbates intestinal inflammation. In an OVX‐induced postmenopausal OP model, dysbiosis of the gut microbiota elevates NLRP3 expression, enhances caspase‐3/8/9 activity and increases IL‐1β release, collectively driving OBs' pyroptosis [153]. Neuropeptide Y, as an upstream regulator, activates the NLRP3/caspase‐1/GSDMD pathway, leading to elevated secretion of LPS, IL‐1β and IL‐18. Subsequently, caspase‐11 directly recognises and binds LPS, stimulating IFN‐γ signalling to trigger OBs' pyroptosis [154]. These findings highlight the role of NLRP3 in modulating the brain‐gut‐bone axis and its involvement in OP pathogenesis. In diabetes‐associated OP models, high glucose induces the transcription of mtROS/NLRP3/caspase‐1 promoting the accumulation of malondialdehyde (MDA) and LDH. This leads to mitochondrial oxidative dysfunction and pyroptosis in OBs. Concurrently, glucolipotoxicity causes overexpression of TLR4, triggering oxidative phosphorylation disruption and increased NLRP3 levels. This upregulates caspase‐3, caspase‐9, TNF‐α and IL‐1β, while inhibiting the expression of BMP2 and Runx2 and the activity of OBs [21, 49]. Furthermore, LPS/ATP treatment induces DDIT3 overexpression, suppressing PINK1/Parkin‐mediated mitophagy and accelerating mtDNA damage. This cascade activates the NLRP3/caspase‐1/GSDMD pathway, triggering OB cleavage, pyroptosis and inflammatory bone degradation [155]. Microgravity is a common mechanical and biological force in the OB surrounding environment. Long‐term exposure can increase the release levels of NLRP3, caspase‐1, GSDMD, IL‐1β and LDH, downregulate the expression of OCN and COL‐I and promote the pyroptosis of OBs [156].

### 5.5 NLRP3 Inflammasome Involvement in Osteoclast Pyroptosis

Bone immune imbalance triggers complex interactions among immune cells and cytokines, driving OCs‐mediated bone resorption, reducing bone mineral density and increasing fracture risk in OP. The NLRP3 inflammasome, however, plays a dual role, directly or indirectly regulating OCs' pyroptosis and contributing to bone homeostasis under physiological conditions. Notably, NF‐κB, a key component of NLRP3 inflammasome assembly, serves as a central signalling pathway in OCs' activation. Studies in OVX‐induced OP mouse models have demonstrated that inflammatory cascades activate NF‐κB‐driven NLRP3 signalling, leading to increased cytoplasmic secretion of TNF‐α, IL‐1β and IL‐18. This enhances RANKL‐induced OCs' proliferation in a concentration‐dependent manner, ultimately resulting in trabecular bone loss and microstructural deterioration [47]. In the inflammatory bone defect model, crosstalk between the NF‐κB and MAPK signalling pathways drives the upregulation of nuclear factor of activated T cells c1 (NFATc1) and IL‐1β, thereby enhancing RANKL‐induced OCs' formation [25]. Conversely, high expression of OPG competitively binds to RANK with RANKL while upregulating NLRP3, Caspase‐1 expression. This process promotes OCs' pyroptosis, facilitating bone repair and remodelling [157]. Simultaneously, it activates the NLRP3‐GSDMD/E‐IL‐1 axis, upregulating IL‐1β and IL‐18 expression, promoting soluble OCs' pyroptosis and counteracting glucocorticoid/ATP‐induced bone defects [158]. Additionally, GSDMD functions as a regulatory checkpoint for lysosomal maturation and secretion, exerting anti‐OCs effects. By restoring lysosomal integrity and maintaining bone homeostasis, GSDMD helps prevent aging/OVX‐induced OP [159]. The implantation of bone materials generates wear particles, triggering inflammation, pyroptosis and osteoporotic osteolysis. Prolonged exposure to these particles leads to ROS accumulation, activating the NLRP3/caspase‐1 pathway in BMMs and upregulating the release of mature IL‐1β and inflammatory mediators. This cascade promotes osteoclast proliferation, migration and bone resorption, exacerbating bone loss [160, 161]. In a titanium particle‐induced calvarial osteolysis model, elevated Ang1 expression triggers mitophagy and engages phosphorylation signalling pathways, thereby inhibiting caspase‐1‐mediated pyroptosis and suppressing LPS/RANKL‐induced OCs' formation [124].

**FIGURE 6:**
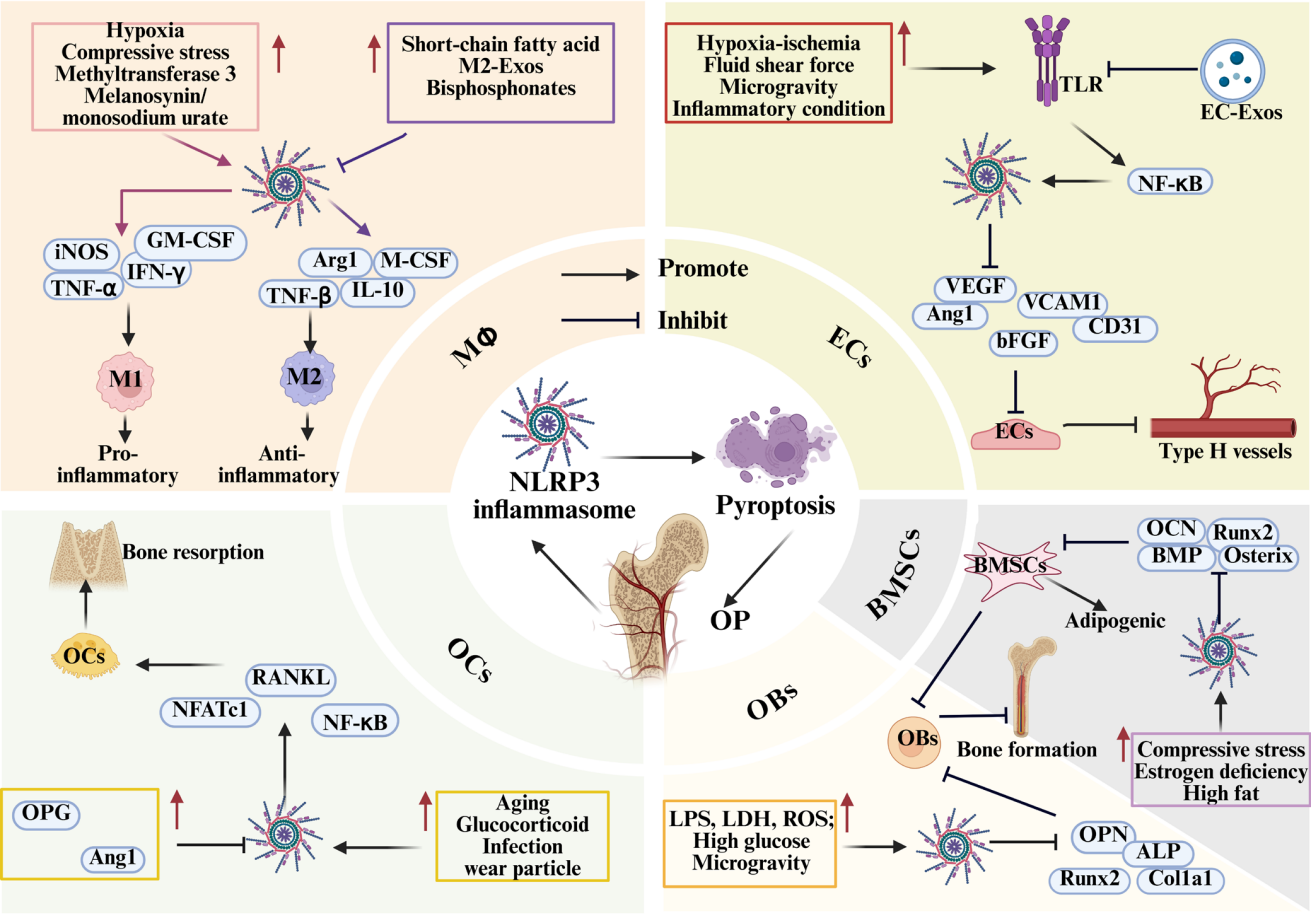
Mechanistic role of NLRP3 inflammasome‐mediated pyroptosis in osteoporosis. Stimulation of the NLRP3 inflammasome by the external environment, chemical substances and ion homeostasis destruction participates in the pyroptosis of macrophages, endothelial cells, BMSCs, OBs and OCs and then participates in the pathological progress of OP.

Additionally, the NLRP3 inflammasome regulates skeletal cells, including osteocytes and fibroblasts, playing a pivotal role in the pathogenesis of OP. Osteocyte pyroptosis, triggered by bisphenol A or wear particles, activates the ROS/NLRP3/caspase‐1 pathway, promoting pyroptosis and bone resorption [48, 162]. In fibroblasts, LPS‐induced NF‐κB activation triggers the NLRP3/caspase‐1/GSDMD signalling axis, upregulating IL‐1β, IL‐6 and IL‐18 to initiate pyroptosis, thereby promoting inflammation and secondary bone erosion in OP [163]. Collectively, targeting key pathways in the pyroptosis of various bone cell types offers new insights into the pathogenesis of OP and provides a theoretical foundation for the development of novel therapeutic strategies (Figure 6) However, significant challenges remain in establishing a clear connection between NLRP3‐mediated pyroptosis and OP, with a notable lack of in‐depth exploration of the underlying mechanisms. Critical steps include elucidating the regulatory pathways that govern NLRP3 inflammasome assembly and activation, as well as the specific mechanisms of pyroptosis, particularly focusing on terminal proteins such as GSDMs. Furthermore, while much of the current research is based on cellular and animal models, large‐scale clinical studies are essential to validate these findings, identify how to precisely control target pathways and genes and translate the insights gained from animal models into clinical practice.

## 6 Targeting NLRP3 Inflammasome‐Mediated Pyroptosis as a Strategy for OP Prevention and Treatment

### 6.1 Exosomes

Exos, as an emerging acellular therapeutic platform, serve as nanocarriers for nucleic acids, lipids and bioactive molecules, facilitating targeted intercellular communication via ligand‐receptor interactions, membrane fusion and endocytosis. By delivering non‐coding RNAs and bioactive factors to pathological sites, Exos modulate NLRP3 inflammasome‐mediated pyroptosis, presenting a promising strategy for preserving bone homeostasis. Notably, Exos derived from BMSCs, endothelial progenitor cells (EPCs) and adipose‐derived stem cells (ADSCs) exhibit high target specificity, superior biocompatibility and low immunogenicity, making them a compelling avenue for osteoprotective interventions [45, 70] (Table 1). However, the multitarget and multi‐pathway effects of Exos give rise to complex crosstalk, necessitating further investigation into the precise mechanisms governing NLRP3 inflammasome assembly. Bridging the gap between preclinical findings and clinical application requires elucidating strategies for enhancing in vivo cell‐specific targeting and efficient uptake, ultimately advancing Exos‐based therapies toward clinical translation.

**TABLE 1:** Exos‐mediated regulation of the NLRP3 inflammasome in osteoporosis: Mechanisms and functional implications.

Source of Exos	Models	Mechanism	Effect	References
BMSCs‐Exos	Tibial bone loss in mice	Activate of miR‐367‐3p/zeste homologue 2 pathway, inhibit NLRP3/caspase‐1/GSDMD axis, ROS, IL‐1, IL‐18 release	Inhibition of EC and OB pyroptosis	[164]
	Spinal cord bone loss in mice	Promote BECN1 and LC3II‐mediated autophagy, inhibit the expression of NLRP3, GSDMD‐N, Caspase‐1 and IL‐1β	Inhibition of BMMs' pyroptosis	[165]
	Knee bone erosion in rats	Inhibition of NLRP3/Caspase‐11/GSDMD axis, downregulation of IL‐1β, IL‐6, TNF‐α, iNOS; upregulated of Arg1, IL‐10 levels	Inhibition of M1 pyroptosis and promote M2 polarisation	[166]
	Ischemia/hypoxia induced bone loss in rats	Inhibition of ROS/TXNIP/NLRP3 axis, downregulation of IL‐1β, IL‐18, TNF‐α; upregulated of VEGF, bFGF levels	Inhibition of BMSCs' and EC pyroptosis	[167]
	Postmenopausal OP women	Inhibition of NF‐κB/NLRP3 axis, Caspase‐8, IL‐1β levels	Inhibition of BMSCs' and OB pyroptosis	[168]
EPCs‐Exos	Diabetic bone defect in rats	Inhibition of NRLP3/caspase‐1/IL‐1β axis, upregulation of VEGF, bFGF, and Ang1 levels	Inhibition of EC pyroptosis	[169]
	High glucose induces human damaged ECs	Inhibition of NLRP3/ASC/caspase‐1 axis, downregulation of IL‐1β, IL‐18, ROS, MDA, LDH levels	Inhibition of EC pyroptosis and oxidative damage	[170]
ECs‐Exos	LPS induced rat calvarial osteolysis	Inhibition of DDX3X/NLRP3 axis, downregulation of IL‐1β and IL‐6, upregulation of IL‐10, Arg1, Runx2, OCN and CD31 levels	Inhibition of EC and OB pyroptosis and promoting M2 polarisation	[137]
ADSCs‐Exos	Diabetic OP in rats	Inhibition of NLRP3/ASC/Caspase‐1 axis, IL‐1β, IL‐18 levels	Inhibition of OC activity	[171]
	Drug‐related jaw loss in mice	Inhibition of NF‐κB/NLRP3/IL‐1β axis, iNOS level	Inhibition of BMMs' pyroptosis, M1 polarisation	[172]
MΦ‐Exos	Tibial femur loss in mice	Promotion of TLR9/NF‐κB/NLRP3 axis, upregulation of IL‐1β level	Promotion of EC pyroptosis and BMSCs' heterotopic ossification	[173]
OCs‐Exos	Lead exposure‐induced OP in rats	Promotion of miR‐30a‐3p/NF‐κB/NLRP3 axis, upregulation of caspase‐1, IL‐1β, IL‐18, GSDMD levels	Promotion of OB pyroptosis	[174]
Serum‐Exos	Knee osteolysis in rats	Inhibition of Sir1/NF‐κB/NLRP3 axis, downregulation of IL‐1β, IL‐18, TNF‐α, LDH levels	Inhibition of BMSCs' pyroptosis	[175]
Liver‐Exos	Diabetic alveolar bone loss patients/mice	Promote NLRP3 palmitic acidification, upregulation of GSDMD, IL‐1β, and fatty acid levels	Promotion of BMSCs' and OB pyroptosis	[176]

### 6.2 Natural Active Monomers

Natural bioactive compounds, characterised by multitarget effects and high safety profiles, modulate NLRP3 inflammasome activation and regulate osteoporotic cell pyroptosis, providing potential therapeutic targets and clinical intervention strategies. Phytochemical monomers – bioactive components isolated from traditional Chinese medicine, including flavonoids, terpenoids and glycosides – exhibit osteometabolic regulatory potential. For instance, certain flavonoids and terpenoids inhibit NLRP3 inflammasome activation, reducing proinflammatory cytokine release while enhancing angiogenic–osteogenic coupling; glycosides, conversely, demonstrate potent osteogenic and immunomodulatory effects, bidirectionally balancing bone homeostasis to ameliorate OP pathogenesis (Table 2). While emerging evidence suggests that certain bioactive compounds may enhance bone mineral density through NLRP3 inflammasome modulation, conclusive proof of their direct engagement with NLRP3 components remains elusive. Future investigations should adopt multidisciplinary strategies – integrating molecular and cell biology with single‐cell sequencing and mass spectrometry – to systematically elucidate the mechanisms of herbal monomers. Current research, predominantly confined to preclinical models and OP animals, lacks clinical validation. Advancing this field requires coupling multi‐omics approaches (e.g., spatial transcriptomics, metabolomics) with multicentre randomised controlled trials, enabling precise dissection of how these compounds target NLRP3 pathway checkpoints, including ASC oligomerisation and Caspase‐1 activation. Future studies should prioritise elucidating synergistic interactions among herbal monomers and assessing their combined efficacy and long‐term safety with established therapies (e.g., bisphosphonates, RANKL inhibitors). Notably, bioactive monomers may orchestrate crosstalk between NLRP3 and multi‐signalling hubs, including ERK/NF‐κB and JAK/STAT pathways. Mechanistic validation demands dynamic interrogation using CRISPR‐based models and intravital imaging to resolve spatiotemporal regulatory dynamics. Such efforts could establish NLRP3‐centric therapeutic frameworks, aiming to refine bone microarchitecture and mitigate fracture risk in OP through precision network modulation.

**TABLE 2:** Mechanisms and functional roles of natural bioactive monomers in modulating the NLRP3 inflammasome in osteoporosis.

Active monomer	Chemical structural formula	Vivo/ex vivo models	Mechanism	Effect
Icariin [177, 178]	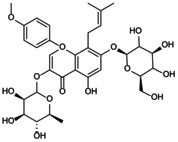	Intestinal microbiota disorder induced OP in mice/Copper sulfate‐induced zebrafish OP	Inhibition of MAPK/NF‐κB/Nod‐like receptor signalling, NLRP3/caspase‐1/IL‐1β axis, downregulation of ROS and TNF‐α levels	Improve intestinal flora, inhibition of OB pyroptosis
Dioscin [179]	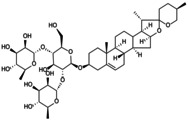	Alveolar bone loss in mice	Inhibition of K^+^ efflux‐mtROS‐mtDNA pathway, NF‐κB/NLRP3/IL‐1β axis, upregulation of Runx2, Osterix, OCN, OPN, ALP levels	Inhibition of OB pyroptosis
Paeoniflorin [180]	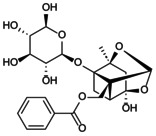	LPS/ATP‐induced pyroptosis of rat macrophages	Inhibition of TLR4/NLRP3/GSDMD axis, downregulation of IL‐18 and IL‐1β levels	Regulation of M1/M2 homeostasis, inhibition of BMMs' pyroptosis
Rutin [181]	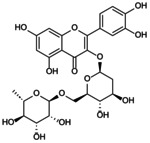	Bone defect in diabetic rats	Inhibition of mtROS/NLRP3/GSDMD axis, upregulate of Arg, IL‐10, Osterix, OPG levels	Inhibition of BMMs, BMSCs' pyroptosis
Forsythoside A [42]	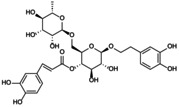	Extraction of BMMs from human femoral defect	Promote mitophagy, Nrf2, LC3II/LC3I signalling; inhibition of NLRP3, Caspase‐1, IL‐1β, IL‐18 levels	Inhibition of BMMs' pyroptosis
Ginsenoside Rh2 [166]	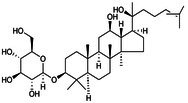	Knee bone erosion in rats	Inhibition of NLRP3/Caspase‐11/GSDMD axis, downregulation of IL‐1β, IL‐6, TNF‐α, iNOS; upregulation of Arg1, IL‐10 levels	Promotion of M1 pyroptosis, M2 polarisation
Astragaloside IV [169, 170]	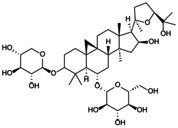	Diabetic bone defect in rats/High glucose induces human damaged ECs	Inhibition of NRLP3/caspase‐1/IL‐1β axis, upregulation of VEGF, bFGF, Ang1; downregulation of ROS, LDH levels	Inhibition of EC pyroptosis
Punicalagin [182]	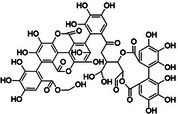	Collagen‐induced osteolysis in mice	Inhibition of NF‐κB/NLRP3/caspase‐1 axis, downregulation of IL‐1β, IL‐18, IFN‐γ, iNOS; upregulation of Arg1, IL‐10 levels	Promotion of M1 pyroptosis, M2 polarisation
Aconitine [68]	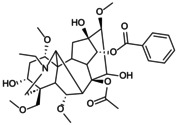	LPS‐induced pyroptosis of mouse BMMs	Inhibition of K^+^, Cl^−^ efflux, inhibition of NLRP3/caspase‐1/IL‐1β axis	Inhibition of BMMs' pyroptosis
Phellodendrine [183]	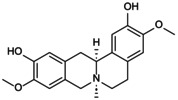	Diabetic OP in rats	Activate the Nrf2/HO‐1 pathway, inhibit the NLRP3/Caspase‐1/IL‐1β/18 axis, upregulate of CD31, VEGF, Osterix, OPG levels	Inhibition of EC and OB pyroptosis
Trehalose [40]	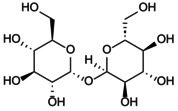	Ovariectomy‐induced OP in postmenopausal mice	Promote autophagy mediated by BECN1 and LC3II/LC3I, inhibition of NLRP3, Caspase‐1, GSDMD‐N, IL‐1β, IL‐18 levels	Inhibition of OB pyroptosis
Quercetin [160, 184]	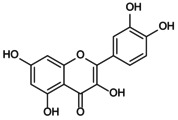	LPS/ATP‐induced pyroptosis of mouse macrophages/Nanoparticle‐induced osteolysis in rats	Activation of Nrf2/HO‐1 pathway, inhibition of NF‐κB/NLRP3, Caspase‐1, IL‐1β, ROS, LDH levels	Inhibition of BMSCs' and OB pyroptosis, Promotion of M2 polarisation
Hecogenin [50]	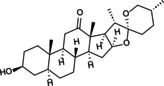	LPS‐induced osteolysis in mice	Activation of Nrf2/HO‐1 pathway, inhibition of NF‐κB/NLRP3/IL‐1β/18 axis, RANKL/NFATc1 pathway, downregulation of LPS, ROS, iNOS, TNF‐α levels, promotion of Arg‐1, SOD levels	Promotion of M2 polarisation, Inhibition of OC formation
Naringenin [56]	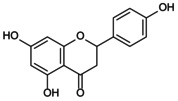	Pyroptosis of mouse OBs induced by microgravity	Activation of Nrf2/HO‐1 pathway, inhibition of microgravity NLRP3/IL‐1β/18 axis, mtROS accumulation, upregulation of ALP, Runx2, BMP2, OPN levels	Inhibition of OB pyroptosis
Luteolin [185]	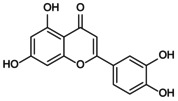	Elderly patients with postmenopausal OP	Inhibition of Notch1/NLRP3 axis, NF‐κB, IL‐1β, IL‐6, IL‐18, TNF‐α levels	Inhibition of EC pyroptosis
Urolithin A [47, 186]	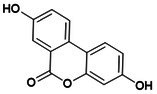	LPS‐induced osteolysis in mice/OCs and postmenopausal OP in ovariectomised mice	Inhibition of AMPK, NF‐κB/NLRP3/Caspase‐1 p20 axis, iNOS, TNF‐α, IL‐1β, IL‐6, NFATc1 expression, upregulation of Arg‐1, IL‐10 levels	Inhibition of BMMs, EC pyroptosis, OC generation, promotion of M2 polarisation
Galangin [187]	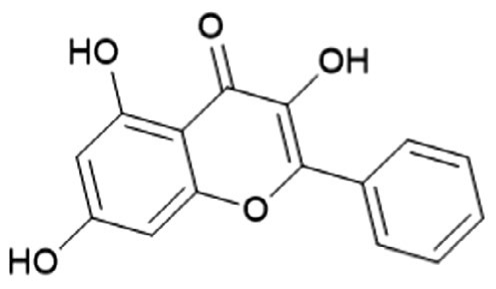	LPS‐induced pyroptosis of rat BMMs	Inhibition of MAPK, NF‐κB/NLRP3/IL‐1β axis, downregulation of TNF‐α, IL‐17, RANKL levels	Inhibition of OC formation
Melatonin [188]	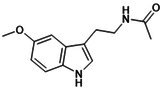	Cadmium‐induced OP in the duck	Inhibition of the Cadmium/NLRP3/Caspase‐1 axis, LDH, IL‐18, IL‐1β levels, upregulation of COL1A, OPG, OPN, Runx2 levels	Inhibition of OC formation, BMMs' and OB pyroptosis
Aloe emodin [109]	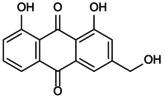	High glucose‐induced mouse ECs	Inhibition of NLRP3 ubiquitination, Inhibition of the NLRP3/ASC/Caspase‐1 axis, upregulation of AngII, VEGF levels	Inhibition of EC pyroptosis
Cucurbitacin B [189]	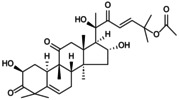	Bone degradation in mice	Activation of Nrf2/HO‐1 pathway, inhibition of microgravity NF‐κB/NLRP3 axis, downregulation of iNOS, IL‐1β, IL‐18 levels	Inhibition of M2 pyroptosis
Celastrol [190]	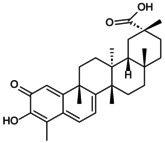	LPS‐induced tendon‐bone injury in rats	Increase the maximum load and stiffness, Inhibition of NLRP3/IL‐1β axis; upregulation of HIF‐1α, VEGF levels	Inhibition of EC and OB pyroptosis
Dicoumarol [191]	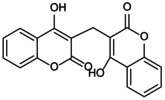	LPS/ATP treatment of rat fibroblasts	Inhibition of mtROS/NF‐κB/NLRP3 axis; downregulation of TNF‐α, COL1A, IL‐1β, IL‐18 levels	Inhibition of fibroblast pyroptosis
Vanillic acid [192]	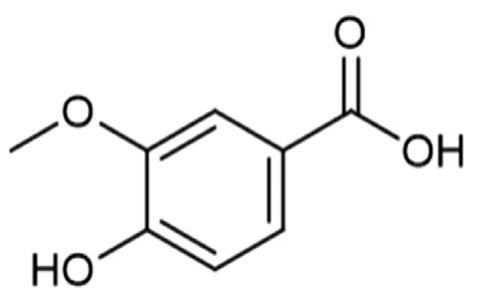	Knee bone erosion in rats	Inhibition of NLRP3/caspase‐1/IL‐1β axis	Inhibition of fibroblast pyroptosis
Rosmarinic acid [193]	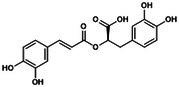	High glucose‐induced diabetic OP in rats	Inhibition of the mtROS/MAPK/NF‐κB/NLRP3 axis, IL‐1β expression	Inhibition of OCs formation, OBs pyroptosis
Ipriflavone [194, 195]	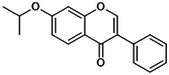	Glucocorticoid‐induced diabetic OP in mice/Early tibial implantation in mice	Inhibition of K^+^ efflux‐mtROS‐mtDNA pathway, NLRP3/caspase‐1/IL‐1β axis	Inhibition of BMMs' and OB pyroptosis
Garcinol [40]	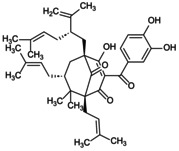	High glucose‐induced EC injury	Inhibition of PI3K/Akt/NF‐κB pathway	Inhibition of EC pyroptosis
Micheliolide [196]	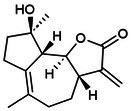	High glucose‐induced inflammatory osteolysis in mice	Inhibition of NF‐κB/NLRP3/capase‐1 axis, downregulation of IFN‐γ, TNF‐α, IL‐1β, IL‐6, IL‐18 levels	Inhibition of BMMs' pyroptosis, Promotion of M2 polarisation
Artesunate [197]	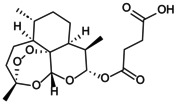	Ligation‐induced alveolar bone loss and RANKL‐induced OCs in mice	Inhibition of RANKL/NLRP3 axis, IL‐1β, IL‐6, TNF‐α expression, upregulation of Runx2, ALP, and OCN levels	Inhibition of OC formation, OB pyroptosis
Cinnamaldehyde [198]	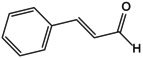	LPS‐induced rat BMMs in vitro	Activation of the tricarboxylic acid cycle, Inhibition of succinic acid/HIF‐1α axis, NLRP3/IL‐1β axis and downregulation of TNF‐α and NO levels	Inhibition of M2 pyroptosis, OC formation
Perillaldehyde [199]	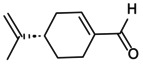	IL‐1β‐treated rat knee bone injury	Enhance mitochondrial autophagy, Inhibition of ALOX5/NF‐κB/NLRP3 axis, downregulate TNF‐α, IL‐6, IL‐8 levels	Inhibition of M2 pyroptosis
Resveratrol [104]	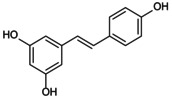	High‐fat diet induced mouse ECs	Inhibition of Sirt1‐p66Shc‐NLRP3 axis downregulates mtROS, LDH levels	Inhibition of EC pyroptosis

### 6.3 Clinical Drug

Current anti‐osteoporotic therapies, spanning anabolic agents (teriparatide, vitamin D, small‐molecule supplements) and anti‐resorptives (bisphosphonates, RANKL‐targeting monoclonal antibodies, oestrogen receptor modulators), counteract OP pathogenesis by directly or indirectly modulating NLRP3 to suppress osteocytic pyroptosis and remodel inflammatory‐immune microenvironments. In addition, clinical hypoglycaemic drugs can be used as NLRP3 inhibitors to prevent diabetic OP and probiotics that regulate the intestinal flora–immune system–bone metabolism axis are widely used. (Table 3) However, most of them indirectly regulate NLRP3 activity and are still not clearly designed for inflammasome inhibition [216]. Therefore, linking these mechanistic insights with current clinical practice will provide more measures for future treatment.

**TABLE 3:** Study on the mechanism and function of clinical drugs regulating Nlrp3 inflammasome in osteoporosis.

Classification	Drug	Models	Mechanism	Effect	References
Bone‐forming drug	Teriparatide	Oestrogen‐depleted mouse OP	Indirect inhibition of NLRP3 signalling, downregulate TNF‐α, IL‐1β and IL‐6 levels	Inhibit OB pyroptosis	[200]
		Immunostimulatory response in vitro in mice	Inhibition of intracellular Ca^2+^ load, downregulate IL‐1β, IFN, IL‐8, TNF‐α, prostaglandins levels	Inhibit BMSCs' and OB pyroptosis	[201]
	Vitamin D	Rat OP induced by high glucose environment	Inhibition of PI3K/AKT/NLRP3 axis, downregulate IL‐1β、IL‐18、TNF‐α levels, upregulate of Runx2, ALP, OPN levels	Inhibit BMSCs' and OB pyroptosis	[202]
		Postmenopausal OP patients	Inhibition of NLRP3 inflammasome polymorphism, downregulate IL‐1β, IL‐18, RANKL levels	Inhibit OB pyroptosis, promote OC necrosis and apoptosis	[8]
Anti‐bone resorption drugs	Bisphosphonates	LPS‐induced BMMs' inflammatory injury	Inhibition of LC3II, BECN1, NLRP3 expression and improvement of BMMs mitophagy	Inhibit BMMs' pyroptosis	[203, 204]
	Denosumab	Postmenopausal OP rats	Downregulation of estradiol, IL‐6 and type I collagen C‐terminal peptide, improves the intestinal‐bone axis	Inhibit BMSCs' and OB pyroptosis	[205, 206]
		Postoperative patients of total hip joint replacement	Indirect inhibition of NF‐κB/NLRP3 axis by reducing TNF9 expression	Inhibit OB pyroptosis	[207]
Hypoglycaemic agents	Glibenclamide	Alveolar bone loss in rats	Inhibition of occlusal stress, NLRP3/IL‐1β, RANKL signal	Inhibit OB pyroptosis	[203, 208]
		High glucose diet induced osteolysis in rats	Improve NLRP3 glycolytic reprogramming, inhibit NLRP3/caspase‐1/IL‐1β axis, upregulate of VEGF levels	Inhibit EC pyroptosis	[209]
		Inflammatory in vitro model	Improve the balance between NLRP3 inflammasome and autophagy flux and inhibit AKT phosphorylation	Inhibit OB pyroptosis	[210]
	Metformin	Inflammatory osteolysis in mice	Inhibition of AKT/mTOR/NLRP3 axis	Inhibit EC, BMSCs and OB pyroptosis	[7, 211]
		Inflammatory bone erosion in mice	Inhibition of NLRP3/caspase‐1/IL‐6 axis	Inhibit BMMs' pyroptosis, promote M2 repolarisation	[212]
		Alveolar bone loss in mice	Inhibition of NF‐κB/NLRP3/Caspase‐1/11 axis, COL1A degradation	Inhibit OB pyroptosis, OC differentiation	[213]
Probiotics	*Lactobacillus plantarum* 45	LPS‐induced bone loss in mice	Improve intestinal flora disorders, inhibition of the Protein tyrosine phosphatase‐2/NF‐κB/NLRP3 axis	Inhibit OB pyroptosis	[214]
	Bifidobacterium Strains	Alveolar bone loss in mice	Inhibition of NLRP3/Caspase‐1/IL‐1β axis, downregulate of TNF‐α, IL‐17 levels	Inhibit BMSCs' and OB pyroptosis	[215]

### 6.4 Small‐Molecule Inhibitors

Advances in protein structure modelling and computational screening have accelerated the identification of potent small‐molecule inhibitors targeting the NLRP3 inflammasome. Given the complexity of its activation pathways, direct NLRP3 inhibitors such as MCC950, CY‐09 and OLT1177 have emerged as leading therapeutic candidates with high specificity and clinical potential for mitigating pyroptosis‐driven pathology. Several NLRP3 inflammasome inhibitors, including MCC950, have progressed to clinical application or early‐phase trials, demonstrating favourable safety profiles. MCC950, a diarylsulfonylurea‐derived small molecule, selectively blocks both canonical and non‐canonical NLRP3 activation by preventing ASC oligomerisation without disrupting NLRP3–NEK7 or NLRP3–ASC interactions. Its non‐covalent binding inhibits NLRP3 ATPase activity and stabilises its inactive conformation, thereby suppressing inflammasome assembly and pyroptosis. MCC950 has shown therapeutic potential in correcting immune dysregulation, including suppression of fibroblast inflammation, MΦ pyroptosis and inflammatory bone loss. However, clinical development was halted during phase II trials due to hepatotoxicity concerns. Structural analogues such as IFM‐2427, inzomelid and somalix are currently under clinical investigation as next‐generation NLRP3 inhibitors with improved safety profiles. The C‐172 analogue CY‐09 competitively binds to the Walker A motif of the NLRP3 NACHT domain, disrupting ATPase activity and selectively blocking inflammasome assembly and activation [217]. Unlike broad‐spectrum inhibitors, CY‐09 does not alter pro‐IL‐1β/IL‐18 expression, ion flux, or mitochondrial function, thereby minimising off‐target effects and preserving cellular homeostasis [218]. Clinical evidence demonstrates that CY‐09 effectively suppresses NLRP3 activation in synovial fluid cells from osteoarthritis patients, leading to reduced IL‐1β and TNF‐α secretion [219]. Orally administered NLRP3 inhibitors – including dapansulene (OLT1177), dimethyl sulfoxide and SN3‐1 – exhibit favourable safety profiles and pharmacokinetics in vivo. These compounds directly target the NACHT domain, inhibit ATPase activity and disrupt inflammasome activation. Notably, daily oral administration of 1000 mg OLT1177 for 8 days in clinical trials resulted in no adverse effects [220], highlighting its translational potential for osteoporotic disease intervention (Table 4). Despite promising advances, concerns remain regarding the target specificity and potential toxicity of current NLRP3 inhibitors. Future efforts should focus on optimising existing or newly identified small molecules by preserving key functional moieties and refining core scaffolds to enhance selectivity and safety, thereby advancing next‐generation NLRP3‐targeted therapies.

**TABLE 4:** The clinical application of small molecule inhibitors regulating Nlrp3 inflammasome in osteoporosis.

Small‐molecule inhibitors	Phase	Clinical model	Mechanism	Effect	References
MCC950	Phase I/II	Human monocyte–macrophage cell line	Inhibition of NLRP3/Caspase‐1/IL‐18 axis, downregulation of LPS levels	Inhibition of BMMs' pyroptosis	[221]
		Patients with type 2 diabetic OP	Inhibition of NLRP3/Caspase‐1/IL‐1β axis	Inhibition of BMMs' pyroptosis	[222]
		patients with periodontitis	Inhibition of NLRP3/Caspase‐1/IL‐18 axis, downregulation of ROS, LDH levels	Inhibition of human fibroblasts' pyroptosis	[223]
		Patients with musculoskeletal comorbidities caused by long‐term smoking	Inhibition of TLR4/NLRP3/Caspase‐1 axis	Prevention of pyroptosis‐induced skeletal muscle atrophy	[224]
CY‐09	Phase II	Osteoarthritis patients	Downregulation of NLRP3, IL‐1β, TNF‐αlevels	Inhibition of BMMs' pyroptosis	[218]
		Umbilical vein endothelial cells induced by myocardial ischemia–reperfusion imbalance	Inhibition of NLRP3/Caspase‐1/GSDMD‐N axis	Inhibition of EC pyroptosis	[219]
Dimethylsulfoxide	Phase I/II	Patients with inflammatory bone defects	Inhibition of TLR4/NF‐κB/NLRP3 axis	Inhibition of BMMs' pyroptosis	[225]
		Human umbilical vein endothelial cell injury	Inhibition of NF‐κB/NLRP3 axis	Inhibition of EC pyroptosis	[226]
OLT1177	Phase II	In vivo implantation of vascularised materials	Inhibition of NLRP3/IL‐1β axis	Inhibit pyroptosis and promote angiogenesis‐osteogenesis coupling	[220]
SN3‐1	Phase I	Rodent model	Inhibition of NLRP3 inflammasome activation	Inhibition of pyroptosis	[227]

### 6.5 Other Avenues

In recent years, bone biomaterials have emerged as key players in the repair and regeneration of osteoporotic bone by modulating immune responses and enhancing the bone microenvironment. Notably, nanomaterials with precise targeting, excellent biocompatibility and controlled release properties have been widely explored for their ability to regulate the assembly of the NLRP3 inflammasome, offering a promising strategy for the prevention and treatment of OP. Studies have shown that cerium oxide nanoparticles modulate MΦ polarisation in a murine model by promoting M2 infiltration and suppressing cathepsin B/NLRP3 signalling. This intervention effectively counteracts LPS‐induced ROS accumulation, mitigates OBs' pyroptosis and enhances the osteogenic microenvironment [228]. In the OVX‐induced bone loss model, the implantation of a nano‐disk co‐loaded with GSK‐J4 targets both MSCs and MΦs, suppressing TLR2/NF‐κB signalling and modulating BMMs epigenetics via the demethylation pathway, reducing the M1/M2 ratio, attenuating NLRP3‐mediated pyroptosis in BMSCs and osteoblasts and serving as a positive feedback mechanism for bone regeneration in OP [229]. The implantation of OBs‐specific aptamer‐functionalised lipid nanoparticles enhances estradiol levels, suppresses NF‐κB/NLRP3/IL‐18 signalling and upregulates Runx2 and OCN expression, a strategy that promotes bone formation, leading to improved bone microarchitecture and mechanical strength [230]. As a bone‐immune homeostasis regulator, graphene oxide nanosheets selectively inhibit the STAT3‐mediated NLRP3/caspase‐1/IL‐1β axis in MΦ, while upregulating RUNX2, Col1a1, CD31 and VEGF expression. This approach promotes H‐type vascular–osteogenic coupling, facilitating osseointegration in OP following OVX [231]. Furthermore, magnetic nanoparticles enhance the bone responsiveness of tissue materials by modulating NLRP3‐mediated bone turnover under low‐grade inflammatory conditions in the bone marrow. This strategy suppresses serum LDH and LPS expression, mitigates senile OP and counteracts bone microstructural deterioration [232], highlighting tissue engineering strategies that target the inhibition of NLRP3 inflammasome expression, opening up a new direction for OP angiogenesis and bone tissue regeneration.

Exercise interventions offer a highly feasible, accessible and cost‐effective approach to OP prevention and treatment. Activities such as walking, resistance training and aerobic exercise have been shown to promote bone regeneration, enhance BMD and effectively mitigate pyroptosis. Studies indicate that moderate‐intensity running can suppress NLRP3/Caspase‐1/IL‐1β signalling in BMSCs, reduce serum levels of TNF‐α and IL‐1β and improve the inflammatory bone microenvironment. These effects contribute to reversing aging‐induced bone loss and tibial microstructural deterioration, highlighting the therapeutic potential of exercise in OP management [233]. Progressive‐load aerobic exercise significantly suppresses the activation of the NLRP3/IL‐1β/IL‐18 pathway in obesity‐associated BMMs, reducing the expression of pyroptotic inflammatory factors. This regulatory effect enhances M2 infiltration and bone remodelling [234]. Additionally, irisin, a myokine released during exercise, has been shown to elevate skeletal irisin levels, suppress NF‐κB/NLRP3/caspase‐1 signalling and protect OBs from high‐glucose‐induced oxidative stress and pyroptotic cell death. In a diabetes‐associated OP mouse model, continuous running training for 8 weeks significantly improved trabecular bone architecture and enhanced bone mechanical properties [235]. In conclusion, a comprehensive exploration of the ‘Exercise‐cell pyroptosis‐OP axis’ is essential for developing novel therapeutic and preventive strategies for OP. However, research on the modulation of OP by exercise via targeting NLRP3 remains in its early stages. In particular, the role of anaerobic exercise in regulating cellular pyroptosis through NLRP3 remains largely unexplored, and the precise mechanisms underlying pyroptosis in exercise models have yet to be systematically elucidated. This knowledge gap hampers the translation of preclinical findings from animal studies to clinical applications, underscoring the need for further investigation.

## 7 Perspectives and Conclusions

The NLRP3 inflammasome, a pivotal component of the innate bone immune system, undergoes aberrant activation in response to external stress, intracellular ions and immune metabolism, driving pyroptotic cell death in MΦ, ECs, BMSCs and OBs. This pathological process disrupts haematopoiesis, bone metabolism and coagulation, ultimately perturbing the angiogenesis–osteogenesis coupling in OP. Elucidating the differential expression of key signalling genes may yield novel biomarkers for early disease detection and monitoring. Emerging evidence suggests that immune response gene 1 and the hypothalamic–pituitary–adrenal axis are central regulators of NLRP3 inflammasome heterogeneity, offering potential therapeutic targets for intervention [236, 237], highlighting the importance of the nervous system in the microenvironment of OP. The triadic regulatory theory of ‘neovascularisation‐osteoblasts‐osteoclasts’ based on OP bone immune repair can be extended to a quadratic regulatory model of ‘nervous system–neovascularisation–osteoblasts–osteoclasts.’ Furthermore, PTM, regulated by protein–protein interactions, stability and subcellular localisation [238], plays a critical role in the precise activation or inhibition of key components within the inflammatory cascade. A recent study identified palmitoylation in sclerotic bone as a key regulator of NLRP3 inflammasome activation and membrane translocation, serving as a counterbalance to excessive immune activation in ischemic and hypoxic environments [101]. These findings provide a microscopic perspective on the evolutionary origins of innate immunity across species and offer a promising avenue for precision‐targeted therapies. However, as current evidence is primarily derived from cellular and animal models, large‐scale clinical trials are essential to validate these mechanisms and their therapeutic potential.

Exos derived from multiple cell sources have emerged as promising regulators of NLRP3 inflammasome expression, modulating MΦ polarisation, enhancing mitochondrial autophagy and maintaining ion homeostasis to inhibit pyroptosis in bone‐related cells. These mechanisms hold significant potential for regenerative medicine and bone tissue engineering in OP. However, this field remains in its early stages, with several critical challenges that warrant further investigation: (1) The diverse cellular origins of Exos contribute to their heterogeneous composition, varying in both quantity and molecular constituents. The specific bioactive components responsible for regulating pyroptosis remain to be fully elucidated; (2) Exos are susceptible to enzymatic degradation and exhibit limited membrane permeability, posing challenges for their therapeutic application. Strategies such as surface modification and reprogramming hold promise for enhancing their regulatory efficacy and represent a compelling avenue for future research; (3) Current evidence suggests that Exos‐mediated regulation of pyroptosis is primarily linked to the NLRP3 inflammasome. However, it remains unclear whether other inflammasomes or non‐canonical pyroptotic pathways can be leveraged for similar regulatory effects. Additionally, while most studies focus on inhibiting pyroptosis, its role may not be exclusively detrimental. Exploring strategies to selectively induce pyroptosis in specific cell types, such as M1 or OCs, could offer novel therapeutic avenues for improving OP prognosis.

Advancements in modern fermentation, isolation and purification techniques have facilitated the development of natural bioactive compounds as potential therapeutics targeting NLRP3 inflammasomes in bone metabolic disorders. However, challenges such as poor stability, low solubility and limited bioavailability hinder their clinical translation. The integration of nano‐delivery platforms offers a promising strategy to enhance the stability and bioactivity of these compounds, thereby mitigating pyroptosis‐induced bone loss and promoting OP bone homeostasis. Wang et al. [239] employed a biomimetic nano‐platform to deliver Puerarin, effectively targeting the NLRP3/caspase‐1/IL‐1β/IL‐18 signalling pathway to suppress pyroptosis in endothelial cells. Additionally, this strategy facilitated the reprogramming of MΦs toward the anti‐inflammatory M2 phenotype, thereby improving the inflammatory microenvironment and synergistically enhancing anti‐pyroptotic effects. Wang et al. [240] developed nanomodified liposomes of Cryptotanshinone to enhance its water solubility. This formulation inhibited the NLRP3/caspase‐1/3 axis, reduced the levels of TGF‐β1, IL‐1β and GSDMD and mitigated oxidative stress and cellular pyroptosis in BMMs, effectively attenuating inflammatory cascade responses. Tang et al. [241] employed colchicine‐loaded nanoparticles to enhance biofilm penetration and promote ordered dispersion. This approach inhibited NF‐κB/NLRP3 signalling, upregulated VCAM1 and VEGF expression and significantly reduced focal ECs death. Xing et al. [181] applied a nanohydrogel loaded with rutin to a diabetic rat fracture model, effectively limiting its off‐target effects. This intervention reduced the release of mtROS and mitochondrial dysfunction and inhibited the pyroptosis‐mediated effects of the NLRP3 inflammasome in BMMs. Therefore, nano‐drug delivery systems enhance the solubility of active compounds, enabling targeted, controlled release and precise drug delivery. However, the development of such systems to promote OP bone healing remains a significant challenge. Future strategies should focus on optimising material shape and surface modifications to improve drug loading capacity for clinical translation. Additionally, the translation into human models presents considerable obstacles. (1) The fabrication of nanocarriers remains complex and presents significant challenges for large‐scale clinical production. Further research is required to optimise preparation methods to enhance their feasibility for clinical application; (2) The diverse physicochemical properties of most Chinese medicine compounds pose significant challenges for nanocarrier‐based co‐loading of multiple therapeutic ingredients. Thus, the development of advanced nano‐delivery systems capable of efficiently encapsulating and co‐delivering multi‐component Chinese medicines is urgently needed; (3) As an emerging drug delivery platform, Exos‐based systems offer advantages such as low immunogenicity, high loading capacity and biodegradability, significantly enhancing targeted delivery. However, the heterogeneity of Exos derived from different sources, particularly variations in membrane surface proteins, presents challenges for standardised screening and therapeutic application. Therefore, further research is needed to develop efficient strategies for the high‐purity isolation of Exos and the precise loading of monomeric therapeutic components.

Previous bioengineered materials were largely designed to disregard the role of the bone immune microenvironment, often leading to suboptimal outcomes. In contrast, NLRP3‐mediated pyroptosis facilitates the transition of nascent biomaterials from an ‘immune‐inert’ to an ‘immune‐regulated’ state, enabling localised, context‐dependent bone regeneration. However, optimising the intensity and timing of NLRP3 inflammasome activation to align with the natural angiogenic‐osteogenic differentiation process remains a major challenge. Addressing this issue is crucial for advancing the clinical translation of bone tissue engineering materials. Ma et al. [242] developed biomimetic hollow nano‐scaffolds incorporating manganese dioxide to target and modulate mitochondrial DNA damage and caspase‐3/GSDME signalling. These scaffolds enabled precise control over photothermal energy conversion, creating a favourable microenvironment for the osteogenic differentiation of BMSCs and facilitating the repair of femoral defects in rats. Wei et al. [243] developed biomimetic nanoplatforms enriched with MΦ membrane shells, enabling site‐specific antioxidant activity and targeted inhibition of the NLRP3/caspase‐1 pathway to mitigate focal injury‐induced cell death. Zhou et al. [83] utilised metallic polyphenol nanoparticles to enhance lysosomal capture and escape capacity, targeting mitochondrial autophagy to clear excessive mtROS and inhibiting GSDMD oligomerisation to degrade NLRP3. Then, greater emphasis should be placed on designing biomimetic materials that incorporate bioactive molecules to modulate inflammatory responses in a controlled manner. Additionally, the development of advanced bone tissue engineering materials should be guided by precisely tailored sequential activation strategies that align with the natural phenotypic transformation during bone healing, offering innovative approaches for regenerative medicine.

Furthermore, given the structural and functional heterogeneity of the NLRP3 inflammasome, future research should leverage advanced techniques such as genomic sequencing and spatial omics to dynamically dissect NLRP3 inflammasome‐mediated pyroptosis [244]. By exploring its spatial heterogeneity, this approach will provide new perspectives on how pyroptosis shapes the bone immune microenvironment and elucidate the communication mechanisms underlying its pivotal role in the bone microenvironment. Additionally, integrating systems‐level modelling will facilitate a deeper understanding of its regulatory role in bone homeostasis, further uncovering the personalised characteristics of OP and providing a theoretical foundation for precision medicine.

Despite substantial progress, insights into the NLRP3 inflammasome's role in OP remain largely restricted to cellular and animal models, with scant characterisation of its structural alterations in human disease. Species‐specific genetic and metabolic differences, alongside artificial induction models and heterogeneity of NLRP3 molecular mechanisms, inadequately replicate the complex human osteogenic microenvironment, impeding clinical translation. To bridge this gap, future studies should employ gene editing and integrated multi‐model platforms – including animal models, organoids and 3D cultures – coupled with advanced bioengineering to improve translational fidelity. Additionally, spatiotemporal mapping of NLRP3 activation during OP progression, leveraging single‐cell transcriptomics and spatial genomics, holds promise for identifying stage‐specific biomarkers and enabling personalised therapies aligned with dynamic bone metabolism. Current treatments mainly utilise oral or subcutaneous delivery, highlighting an urgent need for targeted, stable and safe drug delivery systems. Engineered nanomaterials and hydrogels with controlled release profiles facilitate localised administration of anti‐inflammatory agents, offering transformative potential to modulate the bone microenvironment. Such innovations are poised to enhance therapeutic strategies aimed at promoting angiogenesis–osteogenesis coupling and attenuating pyroptosis‐driven OP pathology.

## Author Contributions

**Jiaxuan Fan:** conceptualization (lead), validation (lead), writing – original draft (lead), writing – review and editing (lead). **Guokai Du:** conceptualization (equal), validation (equal), writing – original draft (equal), writing – review and editing (equal). **Te Ba:** conceptualization (supporting), validation (supporting), writing – original draft (supporting), writing – review and editing (supporting). **HuiXin Sun:** conceptualization (supporting), validation (supporting), writing – original draft (supporting), writing – review and editing (supporting).

## Conflicts of Interest

The authors declare no conflicts of interest.

## Data Availability

Data sharing not applicable to this article as no datasets were generated or analysed during the current study.
